# Goal-directed nutritional support in preserving muscle mass and optimising recovery in treatment of oesophageal cancer: results of a prospective non-randomised cluster trial

**DOI:** 10.1007/s00520-026-11153-4

**Published:** 2026-09-03

**Authors:** Iris Barth, Iris Stelwagen, Linda B. M. Weerink, Dorienke Gort-van Dijk, Hanna Meinders, Milos Milovanovic, Jan Willem Haveman, Marc J. van Det, Gerard Dijkstra, Marjo J. E. Campmans-Kuijpers

**Affiliations:** 1 Department of Gastroenterology & Hepatology, University Medical Centre Groningen (UMCG), Groningen, The Netherlands; 2 Department of Epidemiology, University Medical Centre Groningen (UMCG), Groningen, The Netherlands; 3 Department of Dietetics, University Medical Centre Groningen (UMCG), Groningen, The Netherlands; 4 Department of Radiology, University Medical Centre Groningen (UMCG), Groningen, The Netherlands; 5 Department of Surgical Oncology, University Medical Centre Groningen (UMCG), Groningen, The Netherlands; 6 Department of Surgery, Hospital Group Twente (ZGT), Almelo, The Netherlands

**Keywords:** Nutritional support, Muscle mass, Oesophageal cancer, Bioelectrical impedance analysis, Indirect calorimetry

## Abstract

**Purpose:**

Oesophageal cancer (OEC) treated with neoadjuvant chemoradiotherapy (CRT) followed by oesophagectomy is associated with muscle loss and impaired recovery. We aim to evaluate whether a goal-directed nutritional support protocol (GDNS) is associated with muscle loss and improved survival after CRT and oesophagectomy compared with usual care.

**Methods:**

Adults with OEC undergoing CRT and oesophagectomy were included. In this trial, a tertiary centre provided GDNS, and a secondary hospital offered usual care. Changes in appendicular skeletal muscle index (ASMI) were assessed during CRT and up to 12 months postoperatively using bioelectrical impedance analysis. One-year overall and disease-free survival were investigated.

**Results:**

GDNS and usual care each included 50 patients. Between baseline and CRT completion, ASMI declined in GDNS (-2.53 ± 3.43%) and usual care (-3.20 ± 6.92%, *P* = 0.559), recovered between CRT completion and preoperatively (0.95 ± 4.33 vs 2.51 ± 7.95%; *P* = 0.368; respectively), and declined 12-months postoperatively (-5.75 ± 4.75% vs -7.09 ± 7.38%; *P* = 0.598; respectively). Results remained non- significant after adjustment for confounders and time × group interaction. Survival did not differ between groups.

**Conclusion:**

In both GDNS and usual care, ASMI declines during CRT, recovers after, and decreases postoperatively. The magnitude of ASMI loss is smaller with GDNS than with usual care, although not statistically significant. Survival does not differ between groups. Our findings underscore the need for personalised, integrated interventions to optimise recovery, particularly in the postoperative phase.

**Supplementary Information:**

The online version contains supplementary material available at https://doi.org/10.1007/s00520-026-11153-4.

## Introduction

Globally, oesophageal cancer (OEC) ranks seventh in cancer-related morbidity and sixth in mortality, with higher incidence in men [1]. Currently, the standard of care for patients with locally advanced, resectable OEC is neoadjuvant chemoradiotherapy (CRT) followed by esophagectomy [2, 3]. Although this approach has significantly improved 5-year overall survival (≈48.6%), it is associated with treatment-related morbidity and postoperative complications [4, 5]. Recently, perioperative chemotherapy regimens are increasingly used as an alternative to CRT in this setting [6]. The introduction of minimally invasive and robot-assisted surgery techniques offers advantages over open oesophagectomy [7].

OEC is among the malignancies most strongly associated with unintentional weight loss, driven by dysphagia, reduced calorie and protein intake, fatigue, and an increased resting metabolic rate, all contributing to metabolic derangements and muscle wasting even before substantial weight loss occurs [8]. CRT often exacerbates these losses, reducing treatment tolerance and increasing the risk of further nutritional deterioration [9–11]. Evidence suggests that preoperative nutritional interventions preserve muscle mass and reduce adverse outcomes, suggesting that nutrition-related impairments are reversible when addressed proactively [10, 12]. However, data throughout the entire treatment course, including CRT and the post-operative phase, remain limited.

In response, a goal-directed nutritional protocol (GDNS) was developed using indirect calorimetry to measure energy requirement, monitoring of dietary intake, and close follow-up by a dedicated dietitian. This study aims to investigate whether GDNS can attenuate muscle loss during CRT and up to 12 months after oesophagectomy, compared to usual care. In addition, one-year overall and disease-free survival following CRT and oesophagectomy are evaluated. Finally, post-operative complications, nutritional status, dietary intake, and quality of life (QoL) during CRT and after oesophagectomy are assessed.

## Methods

### Study population and design

Between July 2018 and June 2023, this prospective non-randomised cluster trial (NL6179; NTR6326) included 100 oesophageal cancer patients in two Dutch hospitals: 1) University Medical Centre Groningen (UMCG, intervention/GDNS, enrolment April 2018); 2) Twente Hospital Group (ZGT, usual care, enrolment August 2020).

Different treatment centres were chosen to minimise the risk of the GDNS protocol influencing usual care. Eligible patients were > 18 years with histologically confirmed, previously untreated oesophageal cancer, and scheduled for neoadjuvant CRT and surgery with curative intent. Tumours were located in the upper-, middle-, lower-oesophagus, or oesophagogastric junction. Exclusion criteria were salvage oesophagectomy, cervical lymph-node involvement or metastases, post-cricoid cancer type, Karnofsky Performance Status < 80 or inability to complete questionnaires. The study complied with the Declaration of Helsinki and was approved by the UMCG ethics committee. All patients provided informed consent, including additional consent for the 12-month CT-scan in the GDNS group.

### Data collection

**Fig. 1:**
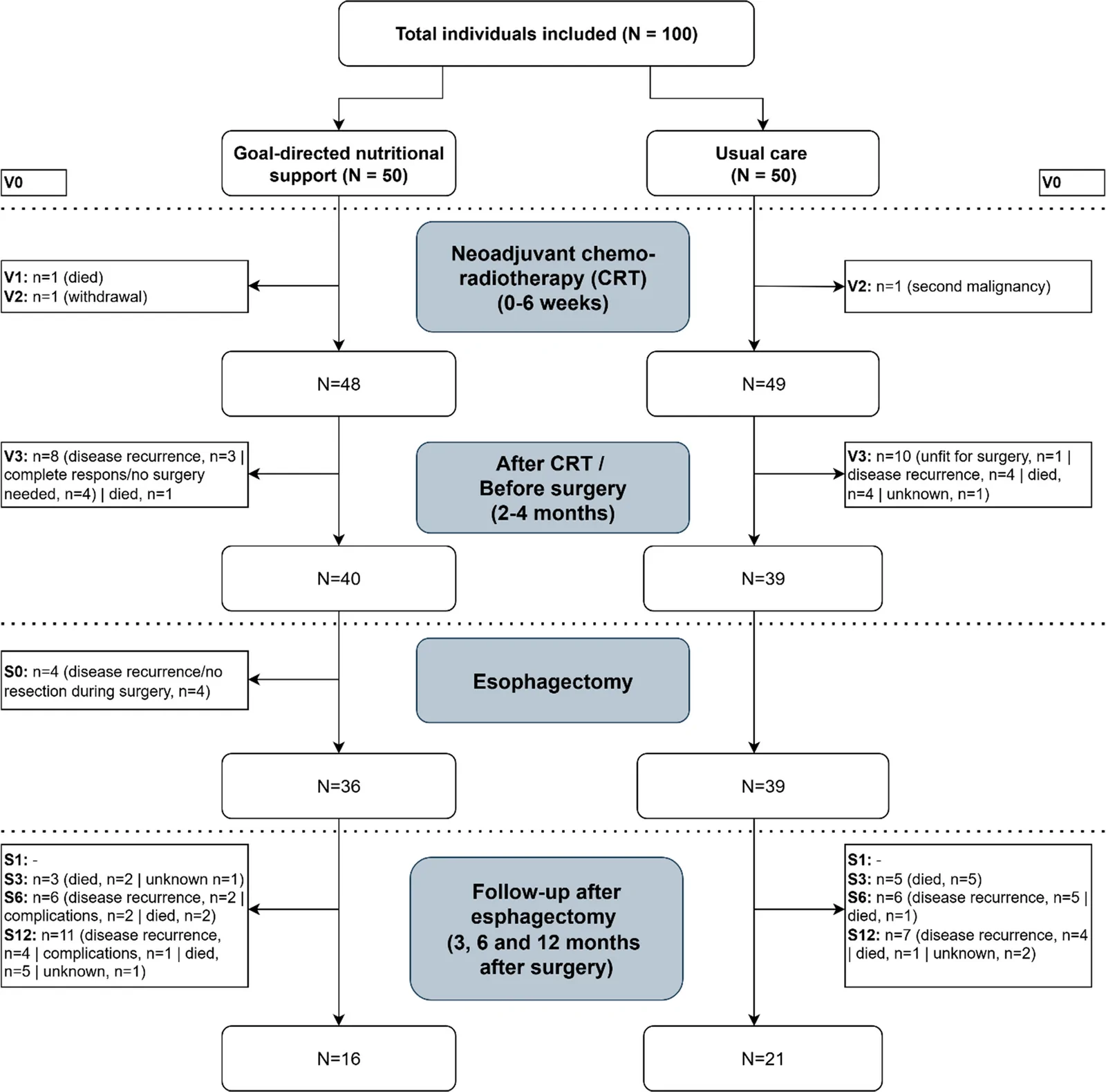
Flow diagram of patient inclusion and lost to follow-up. Abbreviations: CRT, Chemoradiotherapy. V0, baseline visit. V1, 1 week after start CRT. V2, 3–6 weeks after CRT initation. V3, visit between CRT completion and surgery. S0, oesophagectomy. S1, before discharge. S3/S6/S12, 3/6/12 months after surgery

Data were collected at multiple timepoints (Fig. 1): baseline (V0), 1 week after start CRT (V1), 3–6 weeks after CRT initiation (V2), between CRT completion and surgery (V3), oesophagectomy (S0), before discharge (S1), and postoperatively (3, 6, and 12 months; S3, S6, S12). Patient and surgical characteristics were recorded in the data capture tool REDCap [13]. These included: sex, age, body mass index (BMI), educational level, smoking status, alcohol consumption, comorbidities, tumour location and classification, American Society of Anaesthesiology (ASA) classification, surgical procedure type and duration, position of anastomosis, blood loss, postoperative feeding route, length of hospital stay, and resected lymph nodes.

### Intervention and usual care

**Fig. 2:**
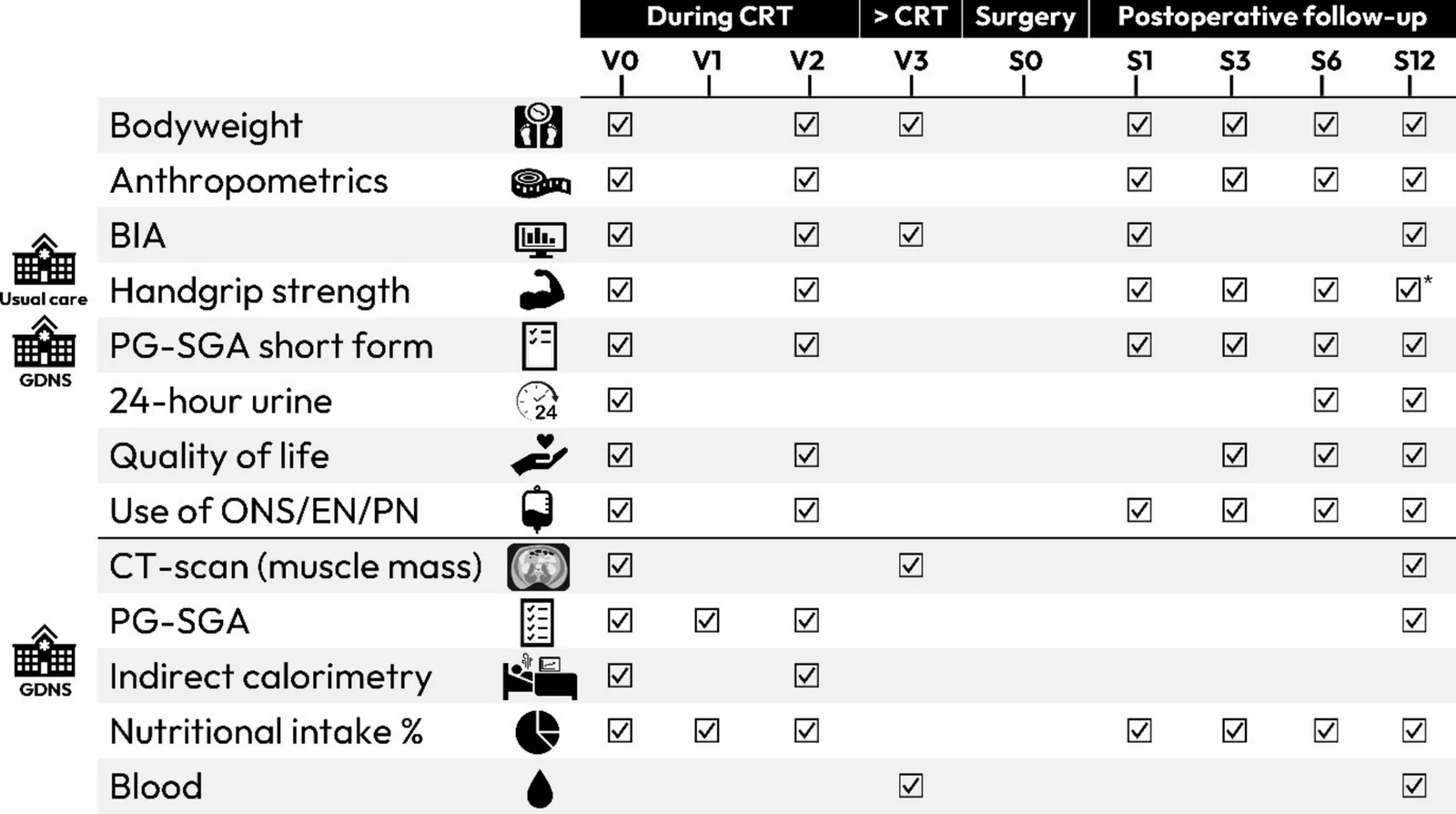
Timeline of the measurement time points and nutritional assessment metrics. Abbreviations: CRT, neoadjuvant chemoradiotherapy. V0, baseline visit. V1, 1 week after CRT initiation. V2, 3–6 weeks after start CRT. V3, visit between CRT completion and surgery). S0, oesophagectomy. S1, before discharge. S3/S6/S12, 3/6/12 months after surgery. BIA, Bio-electrical vector analysis. PG-SGA (SF), Patient-Generated Subjective Global Assessment (Short Form). ONS, oral nutritional supplements. EN, enteral nutrition (tube feeding). PN, parenteral nutrition. *Only for GDNS at this timepoint

In the GDNS group, dietitian and surgical consultations were scheduled concurrently. Throughout the study, patients were assigned a dedicated dietitian (case manager) who monitored nutritional intake and performed all nutritional assessment measurements. Energy requirements were determined with indirect calorimetry (ICal), in addition to standard equations. Furthermore, the Patient Generated-Subjective Global Assessment (PG-SGA) was completed. Nutritional support, including oral nutritional supplements (ONS), enteral nutrition/tube feeding (EN) and parenteral nutrition (PN), was proactively provided (detailed in Supplement 1, methods). In usual care, dietary therapy was provided by different dietitians across multiple locations throughout the study. The dietitian contacted patients during CRT, to monitor weight and food intake, and initiate nutritional support as needed. Unlike the GDNS approach, two oncology research nurses performed nutritional assessment during the full study period except postoperative handgrip strength (HGS), conducted by a physical therapist. Nutritional intake was not routinely recorded, and malnutrition screening was performed using the PG-SGA short form (PG-SGA SF) (Fig. 2).

### Primary outcome

CT-based muscle mass assessment was preferred, but only available for GDNS (V0, V3, and S12), preventing direct comparison with usual care. Therefore, the primary outcome is the difference in relative appendicular skeletal muscle mass index change (∆ASMI), derived from bio-electrical impedance vector analyser (BIA; Seca mBCA 525). Urinary creatinine from 24-h urine collections was included as a surrogate marker for ASMI. Sergi’s formula was applied to obtain ASMI, calculated by dividing ASM by squared height (kg/m^2^) [14]. Relative changes in ASMI at CRT completion (V0-V2), after CRT (V0-V3), and postoperative (V3-S1; V3-S12) were calculated as (current-previous)/previous × 100%. Low ASMI cut-off values were < 7.0 kg/m^2^ (males) and < 5.5 kg/m^2^ (females) [15].

### Secondary outcomes

Abdominal CT images (third lumbar vertebra level) were used to obtain skeletal muscle (SMA) and psoas muscle area (PMA) at V0, V3, and S12, manually outlined with imaging analysis software (Aquarius Intuition, Tera Recon Inc., Foster City, CA, USA). SMA and PMA were divided by squared height (kg/m^2^) to obtain skeletal muscle index (SMI) and psoas muscle index (PMI). Changes in SMI and PMI after CRT (V0-V3) and postoperatively (V3-S12) were calculated as (current-previous)/previous × 100%. Low SMI was defined as: < 41 cm^2^/m^2^ (women), and < 43 cm^2^/m^2^ (men, BMI < 25 kg/m^2^) or < 53 cm^2^/m^2^ (men, BMI ≥ 25 kg/m^2^) [16]. Low PMI cut-off values were 5.65 mm^2^/m^2^ (men) and 4.11 mm^2^/m^2^ (women) [17].

One-year overall (OS) and disease-free survival (DFS) were assessed separately for the CRT and post-operative phases: the time from CRT initiation (or surgery) to death (OS) from any cause, or to disease recurrence or death (DFS). Postoperative complications were reported at S1, S6 and S12: recurrent nerve paresis, pulmonary and cardiac complications, mediastinal abscess, chyle leak and anastomotic leak, delayed gastric emptying, wound infection and dehiscence, reintervention, reoperation, sepsis, ICU readmission, reintubation, organ failure, and death. Complication severity was evaluated at S1 and hospital readmission at S12.

Figure 2 demonstrates the timing of the measurements, detailed in Supplement 1 (methods). Nutritional intake and measured (ICal) resting energy expenditure (REE) were reported for the GDNS group. For both GDNS and usual care, outcomes included: predicted total energy expenditure (TEE), nutritional support (type, quantity), fat free mass (FFMI) and fat mass index (FMI), phase angle (PhA), BMI, waist circumference (WC), triceps skinfold thickness (TSF), mid-upper arm muscle circumference (MAMC), and HGS. Malnutrition assessment included the Global Leadership Initiative on Malnutrition (GLIM) and the Patient Generated-Subjective Global Assessment (PG-SGA, only in GDNS) and short form (PG-SGA SF). Disease specific QoL was assessed with the 30-item European Organization for Research and Treatment for Cancer Quality of Life Questionnaire (EORTC-QLQ-C30) [18].

### Sample size, statistical analysis

We considered a 5% muscle mass improvement during CRT to be clinically relevant, requiring a sample size of 84 patients (power = 80%, α = 0.05, standard deviation = 1.61 cm^2^/m^2^) [19, unpublished UMCG data research B. Rasul 2014]. An additional 15% was included to account for expected variability in outcome distribution, resulting in 100 patients.

Continuous data are presented as mean with standard deviation (SD), median with interquartile range (IQR), and categorical data as frequencies (n) with percentages (%). Group differences (GDNS vs usual care) in outcomes were evaluated with the independent t-test, Mann–Whitney U, or Fisher’s exact test, and within-group changes with a paired t-test, Wilcoxon signed rank, or McNemar test. Analyses (R, version 4.3.3) consisted of a per protocol-approach and two-sided statistical tests (*P* < 0.05 statistically significant) [20].

Correlation between BIA-ASMI and both CT-SMI and urinary creatinine were examined. Analysis of covariance (ANCOVA) was conducted to investigate the association between groups and the outcome (∆ASMI): V0-V2 (at CRT completion), V0-V3 (after CRT/preoperatively), and V3-S12 (after oesophagectomy). Sex, age, smoking, BMI, baseline nutritional assessment, surgical, and complication differences were included as covariates (with a maximum of four) and considered confounding in case of altering treatment effect by ≥ 10%. To account for varying measurement timing, ∆BIA-ASMI (V0–V3) between GDNS and usual care was additionally analysed using generalised linear mixed-effects models. Time (weeks) was entered as a mean-centred continuous variable.

Model selection proceeded from a random-intercept model to adding a random slope, fixed time effects, and quadratic terms. A group × time interaction and covariates were included in the final model. Survival probabilities were estimated with the Kaplan–Meier method and compared between groups (log-rank test). Patients alive and disease-free at the end of follow-up or lost to follow-up were censored at week 52.

## Results

### Inclusion, patient characteristics

Fifty patients were included per group (Fig. 1). Oesophagectomy was performed in 36 (GDNS) and 39 (usual care), while 16 and 21 patients completed the 12-month follow-up, respectively. Median age was 66.0–68.5 years and most patients were male (72–84%). Tumours were located predominantly in the lower thoracic oesophagus (≥ 50.0%) and classified as adenocarcinoma (≥ 82.0%) (Table 1). The GDNS group demonstrated longer surgical procedure time (474 ± 84.1 vs 390 ± 61.6 min, *P* < 0.001), hospital stay (13 [9, 17] vs 7 [7, 8] days, *P* < 0.001), greater blood loss (110 [70, 200] vs 100 [73, 100] ml, *P* = 0.039), and prolonged use of a nasogastric tube (7 [6, 11.5] vs 4 [3, 5] days; *P* < 0.001) compared to the usual care group (Supplement 2, Table S1).

**Table 1:** Patient and disease characteristics

**Demographics**	**GDNS (n = 50)**	**Usual care (n = 50)**
Sex, male	36 (72.0)	42 (84.0)
Age [years]	66.0 [57.0, 71.0]	68.5 [62.0, 73.0]
Body mass index [kg/m^2^]	26.7 ± 4.2	26.2 ± 4.2
Educational level		
Primary	4 (8.0)	2 (4.0)
Lower secondary	19 (38.0)	18 (36.0)
Upper secondary	10 (20.0)	13 (26.0)
Higher or academic	9 (18.0)	17 (34.0)
*Missing*	*8 (16.0)*	*0*
Smoking		
Never	9 (18.0)	13 (26.0)
Former, quit ≤ 10 years	16 (32.0)	12 (24.0)
Former, quit 11–20 years	8 (16.0)	7 (14.0)
Former, quit > 20 years	12 (24.0)	16 (32.0)
1–15 cigarettes per day	2 (6.0)	1 (2.0)
16–25 cigarettes per day	1 (2.0)	1 (2.0)
Alcohol consumption		
Never	18 (36.0)	15 (30.0)
1–2 glasses per month	9 (18.0)	5 (10.0)
1–2 glasses per week	12 (24.0)	15 (30.0)
1–2 glasses per day	4 (8.0)	9 (18.0)
2–4 glasses per day	5 (10.0)	3 (6.0)
4–8 glasses per day	2 (4.0)	1 (2.0)
> 8 glasses per day	0 (0.0)	1 (2.0)
Comorbidities		
Myocardial infarction	5 (10.0)	3 (6.0)
Congestive heart failure	1 (2.0)	1 (2.0)
Peripheral artery disease	4 (8.0)	5 (10.0)
Cerebrovascular accident	3 (6.0)	2 (4.0)
Chronic obstructive pulmonary disease	3 (6.0)	3 (6.0)
Connective tissue disease	0 (0.0)	1 (2.0)
History of stomach ulcer	1 (2.0)	0 (0.0)
History of uncomplicated diabetes mellitus	8 (16.0)	8 (16.0)
History of kidney disease	1 (2.0)	0 (0.0)
History of liver disease	1 (2.0)	0 (0.0)
History of solid tumour	10 (20.0)	3 (6.0)
History of aids	0 (0.0)	1 (2.0)
**Disease characteristics**	**GDNS (n = 50)**	**Usual care (n = 50)**
Tumour location		
Upper thoracic	0 (0.0)	0 (0.0)
Middle thoracic	2 (4.0)	5 (10.0)
Lower thoracic	26 (52.0)	25 (50.0)
Gastro-oesophageal junction	8 (16.0)	9 (18.0)
*Missing*	*14 (28.0)*	*11 (22.0)*
Histological		
Squamous cell carcinoma	5 (10.0)	8 (16.0)
Adenocarcinoma	45 (90.0)	41 (82.0)
Other	0 (0.0)	1 (2.0)

Continuous variables are presented as mean ± SD or median [IQR]; categorical variables as numbers with frequencies (n, %). Abbreviations/symbols: GDNS, goal-directed nutritional support (intervention)

### Primary outcome: ASMI

Correlation between ASMI and urinary creatinine was good (V0: ρ 0.58, *P* < 0.001; S12: ρ 0.72, *P* < 0.001). Within the GDNS group, urinary creatinine remained stable between V0-V6 (11.7 [9.20, 14.9] to 10.8 [9.65, 13.3] mmol/24 h, *P* = 0.201), but declined in usual care (12.5 [10.7, 15.8] to 11.5 [9.60, 12.9] mmol/24 h, *P* = 0.029), demonstrating a significant between-group difference (*P* = 0.028).

**Fig. 3:**
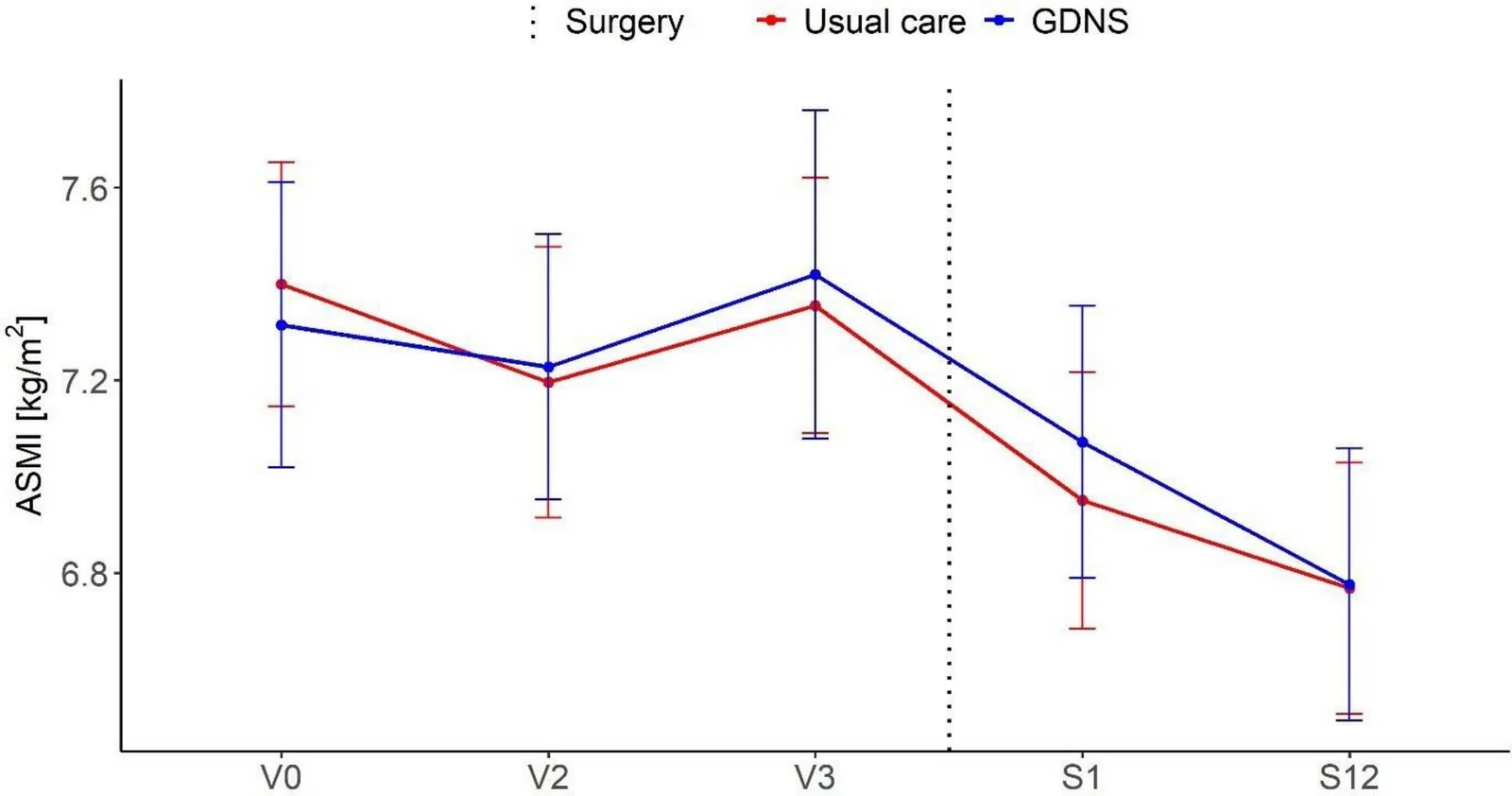
Appendicular skeletal muscle index at each timepoint during the study. Values are presented as mean with standard deviation. Abbreviations: GDNS, goal-directed nutritional support. ASMI, appendicular skeletal muscle index (calculated with Sergi’s formula [14], based on bio-electrical vector impedance analysis derived muscle mass measurements). V0, baseline visit prior to CRTV0, baseline visit. V1, 1 week after start CRT. V2, 3–6 weeks after start CRT. V3, visit between CRT completion and CT surgery). S1, early postoperatively. S12, 12 months after surgery

Between V0-V2, ASMI declined in both usual care (−3.20 ± 6.92%) and GDNS (−2.50 ± 3.43%), and between V0-V3 (−0.38 ± 7.18 vs −1.57 ± 4.05). Preoperatively (V2-V3), ASMI recovered (usual care: 2.51 ± 7.95 vs GDNS: 0.95 ± 4.33; *P* = 0.368). However, peri-operatively (V3-S1), ASMI showed a greater decline in usual care than GDNS (−4.88 ± 5.75% vs −0.93 ± 4.42%, *P* = 0.038). After oesophagectomy (V3-S12), ASMI decreased with −7.09 ± 7.38% in usual care and −5.75 ± 4.75% in the GDNS group. None of these analyses reached statistical significance. Low SMI prevalence did not differ between groups (Table 2, Fig. 3).

**Table 2:** Appendicular skeletal muscle index (ASMI) measurements across the study period

**During CRT**	**n**	**Usual care**	**n**	**GDNS**	***P*-value**	**95%CI**
**V0**						
Low ASMI	50	8 (16.0)	41	6 (12.0)	1.00	
ASMI, all [kg/m^2^]		7.40 ± 0.91		7.31 ± 1.07	0.691	−0.34 to 0.50
ASMI, females		6.47 ± 0.96		6.02 ± 0.95	0.345	−0.54 to 1.44
ASMI, males		7.58 ± 0.80		7.68 ± 0.78	0.579	−0.47 to 0.27
**V2**						
Low ASMI	48	13 (26.0)	37	6 (12.0)	0.298	
ASMI, all		7.00 ± 1.01		7.23 ± 0.99	0.883	−0.47 to 0.40
ASMI, females		5.98 ± 0.90		6.00 ± 0.97	0.970	−1.07 to 1.04
ASMI, males		7.44 ± 0.85		7.52 ± 0.76	0.696	−0.46 to 0.31
∆ASMI V0-V2 [%]	48	−3.20 ± 6.92	35	−2.53 ± 3.43	0.559	−2.98 to 1.62
**V3**						
Low ASMI	33	5 (10.0)	21	1 (2.0)	0.386	
ASMI, all		7.36 ± 0.96		7.42 ± 1.23	0.839	−0.71 to 0.58
ASMI, females		6.29 ± 0.73		5.42 ± 0.78	0.135	−0.36 to 2.10
ASMI, males		7.55 ± 0.87		7.89 ± 0.73	0.164	−0.84 to 0.15
∆ASMI V0-V3 [%]	33	−0.38 ± 7.18	20	−1.57 ± 4.05	0.442	−2.84 to 3.29
**After oesophagectomy**	**n**	**Usual care**	**n**	**GDNS**	***P*-value**	**95%CI**
**S1**						
Low ASMI	30	10 (25.6)	19	3 (8.3)	0.205	
ASMI, all		6.95 ± 0.96		7.07 ± 1.02	0.680	−0.71 to 0.47
ASMI, females		5.43 ± 0.56		5.57 ± 1.27	0.874	−2.87 to 2.60
ASMI, males		7.18 ± 0.78		7.35 ± 0.71	0.472	−0.64 to 0.30
∆ASMI V3-S1 [%]	27	−4.88 ± 5.75	10	−0.93 ± 4.42	0.038^*^	−7.65 to −0.24
**S12**						
Low ASMI	20	7 (12.5)	13	5 (13.9)	1.000	
ASMI, all		6.77 ± 0.94		6.78 ± 1.02	0.983	−0.73 to 0.72
ASMI, females		5.75 ± 0.95		5.22 ± 0.62	0.465	−1.43 to 2.49
ASMI, males		7.02 ± 0.77		7.06 ± 0.80	0.907	−0.68 to 0.60
∆ASMI V0-S12 [%]	20	−6.91 ± 4.82	13	−6.92 ± 4.50	0.996	−3.38 to 3.39
∆ASMI V3-S12 [%]	18	−7.09 ± 7.38	5	−5.75 ± 4.75	0.598	−6.61 to 3.93

Continuous data are presented as mean ± standard deviation or median[interquartile range], and categorical data as frequencies (N) with percentages (%)

Abbreviations: CRT, chemoradiotherapy. GDNS, goal-directed nutritional support protocol (intervention). 95%CI, 95% confidence interval. V1, visit 1 (1 week after start CRT). V2, visit 2, end of CRT (3–6 weeks after start CRT). V3, visit 3 (2–4 months after start CRT/CT before surgery). S1, early postoperatively (during hospital admission/before discharge). S12, 12 months after surgery. n =, number of observations. ASMI, Appendicular Skeletal Muscle mass index [kg/m^2^]; calculated based on Sergi’s formula [14], where ASM was divided by squared height to assess ASMI. Δ, change in ASMI between two timepoints

The potential confounding effect of surgical variables, including blood loss, length of stay, and duration of surgery were investigated with ANCOVA analyses. A sensitivity analysis was performed including sepsis as well. Both analyses demonstrated no significant difference in relative ∆ASMI between GDNS and usual care across the CRT and postoperative period (Table S2-C and S2-D, respectively). In the mixed-effects analysis, a random-intercept model with a quadratic time term best fitted the non-linear ASMI trajectory with a mean time interval (V0-V3) of 17 weeks. The final model demonstrated no intervention effect on ASMI (Table S3, Figure. S1).

### Secondary outcomes

#### CT-muscle

**Fig. 4:**
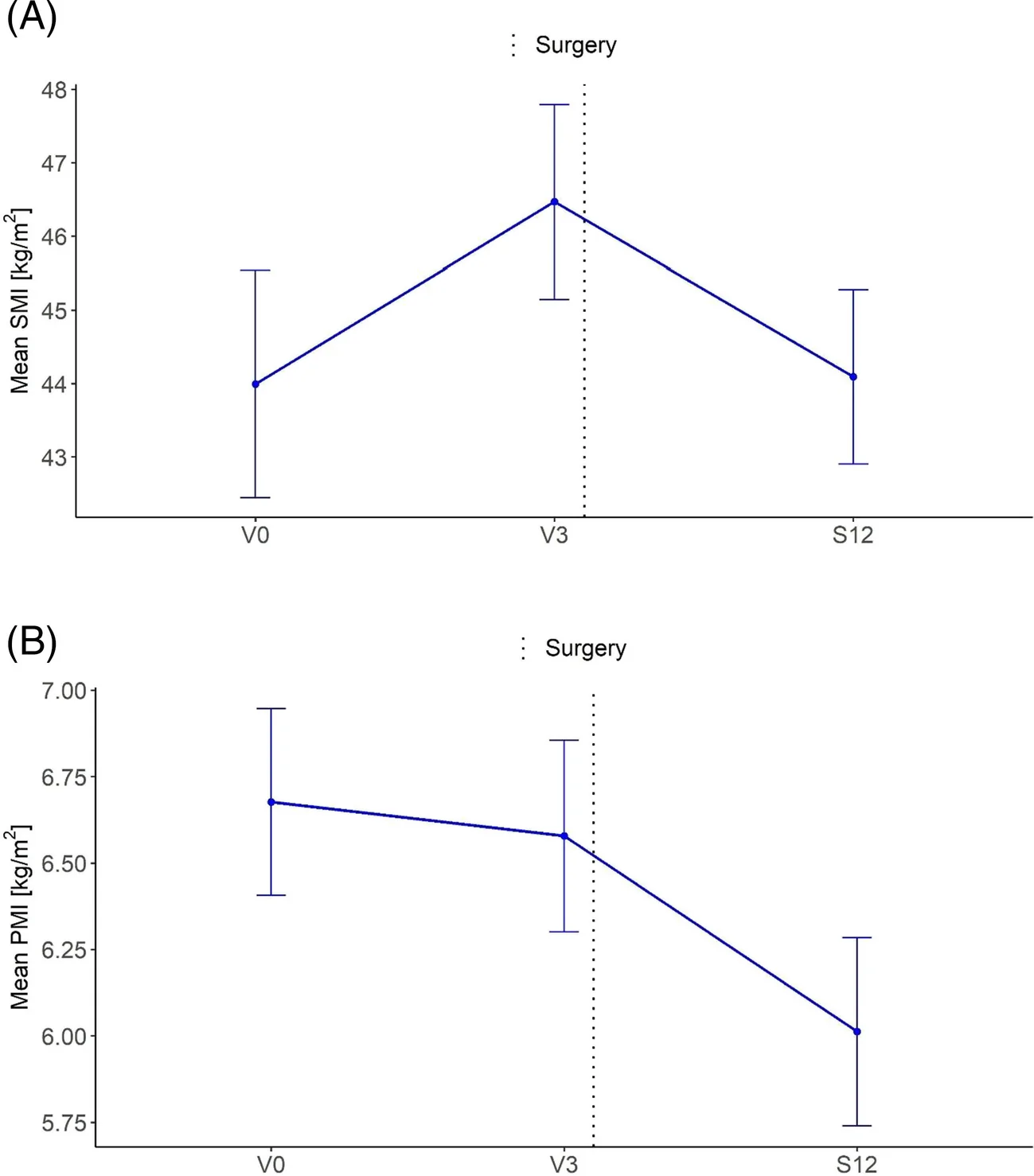
Computed-tomography derived skeletal muscle index **A** and psoas muscle index **B** in the goal-directed nutritional support group. Abbreviations: SMI, skeletal muscle index. PMI, psoas muscle index. V0, baseline visit prior to CRT. V3, visit 3 (2–4 months after CRT initiation. S12, 12 months after surgery

BIA-ASMI correlated significantly with CT-SMI at V0 (*r* = 0.48, *P* = 0.003), V3 (*r* = 0.81, *P* = 0.002) and S12 (*r* = 0.96, *P* < 0.001). SMI did not significantly change between V0-V3, but declined between V3-S12 (−6.21%, 95%CI −11.8 to −0.63, *P* = 0.032). Low SMI was relatively high (≥ 56%) across all timepoints, whereas low PMI occurred less frequently (16.0–40.0%). PMI decreased postoperatively, although not significantly (Fig. 4; Table S4).

#### Survival, postoperative complications

One-year OS after CRT initiation was 88.0% (95% CI, 79.4–97.5) in GDNS and 84.0% (95% CI, 74.4–95.0) in usual care, while one-year DFS was 66.0% (95%CI, 54.1 to 80.5) and 69.7% (95%CI, 58.0 to 83.8), respectively. Postoperative one-year OS was 75.0% (95%CI, 62.1 to 90.6) in GDNS and 82.1% (95%CI, 70.8 to 95.0) in usual care, with corresponding DFS rates of 58.3% (95%CI, 44.3 to 76.9) and 58.9% (95%CI, 45.4 to 76.6). Survival did not differ significantly between groups (Figure S2). Overall complication rates were similar between GDNS and usual care, except for higher sepsis in GDNS (19.4% vs 2.6%, *P* = 0.022) (Table S5).

#### Nutritional support and assessment

##### GDNS group

Between V0-V2, energy intake increased (23.4 ± 8.06 to 28.0 ± 7.9 kcal/kg, *P* = 0.008). Protein intake increased between V0-V2 as well (1.03 ± 0.37 to 1.32 ± 0.43 g/kg, *P* = 0.008), while it decreased from S3 to S6 (99.8 ± 27.5 to 75.8 ± 28.6 g, *P* = 0.022). ICal was performed in 88% (44 of 50) patients at V0 and 72.0% (36 of 50) at V2, yet in only 22.0% (11 of 50) and 37.5% (18 of 48) of cases the measured energy targets were applied in clinical practice, respectively. Measured REE (ICal) was higher at V0 compared to predicted REE (1854 [1703, 2060] vs 1674 [1426, 1820] kcal, *P* < 0.001), similarly to V2. Between V0-V2, measured REE remained stable (Table S6).

##### GDNS and usual care

At CRT completion (V2), the quantity of EN was higher in GDNS compared to usual care (2000 ml [1800, 2000] vs 1500 ml [1250, 1590], *P* = 0.010). Fewer patients used early oral feeding (day 3 postoperatively) in GDNS than in usual care (0% vs 94.6%, *P* < 0.001), and postoperative (S1) ONS use in GDNS was lower than in usual care (9.1% vs 94.1%, *P* < 0.001) whereas quantity of EN was higher in GDNS (1500 ml [1000, 2000] vs 1000 ml [1000, 2000], *P* < 0.001) (Table S7).

During CRT (V0, V2), GDNS demonstrated higher values for TSF, PhA, and HGS than usual care. In both groups, nutritional status declined, with reductions in BMI, SMM, FFMI, and PhA. Additionally, WC and MAMC reduced in usual care. BMI loss between V0 and V2 was smaller in GDNS than in usual care (−0.57 ± 0.85 vs −1.01 ± 0.84 kg/m2, *P* = 0.015). Both WC and FMI increased in GDNS, while it reduced in usual care (0.84 ± 5.72 vs −2.10 ± 2.64 cm, *P* = 0.009; and (0.20 [−0.15, 0.95] vs −0.30 [−1.32, 0.42] kg/m^2^, *P* = 0.017). After CRT completion (V2-V3), BMI improved in both groups, but PhA declined in GDNS and not in usual care (−0.41 ± 0.50 vs 0.09 ± 0.72, *P* = 0.011). After surgery (V3-S1), usual care showed reductions in BMI, SMM, FFMI, and FMI, while PhA decreased in both groups. Long-term follow-up (S1-S12) showed persistent BMI reduction in both groups, and WC reduction in GDNS (Table S8). PG-SGA SF scores increased during CRT (V0-V2) in both GDNS (5.1 ± 4.9 to 7.9 ± 5.9, *P* = 0.001) and usual care (4.2 ± 3.5 to 8.1 ± 5.4, *P* < 0.001). Postoperatively (S1-S12), PG-SGA decreased in GDNS (9.2 ± 3.7 to 4.6 ± 2.8, *P* = 0.008). GLIM-defined malnutrition increased during CRT (V0-V2) in both GDNS (34.1% to 58.5%, *P* = 0.003) and usual care (35.4% to 85.4%, *P* < 0.001). Sarcopenia, occurred in only a few cases (Table S9).

#### Quality of Life

QoL worsened during CRT but recovered after oesophagectomy in both groups (Figure S3). Overall outcomes were better in GDNS compared to usual care, with higher QoL at V2 than in usual care (75.0 [58.3, 83.3] vs 66.7 [41.7 to 75.0] %, *P* = 0.007).

## Discussion

This study demonstrates that in both GDNS and usual care, ASMI decreases during CRT, recovers afterward, and subsequently declines following oesophagectomy. These reductions are less pronounced in GDNS than usual care during CRT (−2.50% vs −3.20%), and 12 months postoperatively (−5.75% vs −7.09%), although statistical significance is not reached. Results remain stable after adjustment for confounders or time × group interaction.

Both CT-SMI and CT-PMI significantly decrease postoperatively in the GDNS group. Survival rates do not differ between GDNS and usual care, while postoperative sepsis rate is higher in GDNS. Nevertheless, GDNS was associated with more favourable outcomes in nutritional intake, body composition, and quality of life compared with usual care.

### Comparison to other studies

Previous studies have demonstrated substantial skeletal muscle loss in patients with oesophageal cancer undergoing neoadjuvant therapy. A meta-analysis demonstrated a pooled muscle loss proportion of −6.69% during CRT in three studies with 397 patients, mean ages of 63.5, 64.6, and 63 years, respectively [19, 21–23]. The authors of the meta-analysis identified both CRT and older age as risk factors for muscle loss during neoadjuvant therapy.

In comparison, the observed muscle loss during CRT in the present study was relatively modest (−2.50% in GDNS and −3.20% in usual care), while the median age was slightly higher (GDNS: 66 years; usual care: 68.5 years).

Our observed muscle loss in patients receiving CRT is comparable to that reported in patients receiving neoadjuvant chemotherapy alone (−2.93%). Although the meta-analysis did not evaluate nutritional interventions and primarily described changes in muscle mass across treatment subgroups, the lower observed muscle loss in the present study may suggest a potential contribution of both the GDNS intervention and usual care.

Nutrition intervention studies have reported beneficial effects of multidisciplinary nutritional management on nutritional status, while systematic enteral nutrition has been associated with reduced weight loss, smaller declines in serum albumin levels, and improved infection rates and survival [24, 25]. Furthermore, *Reijneveld* et al. observed a decline in nutritional status during chemoradiotherapy, followed by recovery during a prehabilitation period including combined exercise training and dietetic counselling before surgery [26]. Although these studies varied considerably in design, intervention type, and outcome measures, their findings consistently suggest that nutritional status can be improved or preserved through targeted nutritional interventions during neoadjuvant treatment for oesophageal cancer, in line with the results of the current study. However, none of these studies specifically evaluated the effect of nutrition on changes in skeletal muscle mass. *Kita* et al. showed that enteral nutrition administered during neoadjuvant chemotherapy was more effective than parenteral nutrition at preventing skeletal muscle loss in patients with oesophageal cancer, with baseline muscle mass, prognostic variables, and nutritional adequacy (kcal) balanced between groups [27].

Following oesophagectomy, two studies reported a postoperative SMI decrease of −6.2% after 6 months, and −5.6% after 4 months [28, 29]. Our observed muscle loss was relatively modest compared to previous reports, since our postoperative measurements were obtained after one year rather than earlier timepoints in those studies.

The present study demonstrated a higher pre-operative CT-SMI compared to baseline, in contrast to BIA-ASMI. This discrepancy may be explained by differences in measurement timing, since we were unable to assess SMI directly at CRT completion. The observed postoperative SMI decline is in line with ASMI reductions in our study and in previously mentioned research [28, 29]. PMI declined notably after surgery, consistent with prior findings [30]. However, between baseline and the pre-operative time point, PMI decreased, while SMI increased. While the choice between SMI and PMI as indicators of muscle mass is debated, the correlation between muscle mass and urinary creatinine levels in the present study supports our findings [31, 32].

An observational study without nutritional intervention demonstrated similar weight and FFMI reductions compared to our usual care group [11]. Weight loss during CRT occurred in the study by *Reijneveld* et al. as well, but improved after following the prehabilitation program [26]. Likewise, another trial reported a smaller BMI decline after a nutritional intervention [33]. Postoperatively, BMI, FFM, and FM declined, in line with an exercise intervention study which found greater postoperative weight and FM loss than controls [34]. The authors report that resistance training preserved FFM, but further improvement was prevented by reduced postoperative protein intake, which was also observed in the present study. Variability in body composition changes may reflect postoperative nutritional challenges, inflammatory responses to surgical stress, in addition to differences in nutritional management [26].

One intervention study reported better compliance with energy targets than controls, whereas another demonstrated reduced energy and protein intake in usual care [11, 33]. In the current study, energy and protein intake improved during CRT in the GDNS group, and EN was higher after CRT than in usual-care. Nutritional decline was less pronounced in GDNS, consistent with evidence supporting nasogastric feeding during CRT [35].

ERAS guidelines recommend early postoperative enteral nutrition, although the optimal route has not been clearly defined [36]. Subsequent studies have shown that early oral nutrition does not affect anastomotic leakage rates, and may offer long-term survival benefits [37, 38]. Early postoperative oral feeding was less common in GDNS than in usual care, suggesting that early oral feeding had already been implemented in usual care (2020) but not yet in GDNS (2018). Overall, we observed that nutritional intake in the GDNS group (23.4–28.8 kcal/kg and 1.03–1.32 g/kg) aligns closely with guidelines (25–30 kcal/kg, ≥ 1.0–1.5 g protein/kg) [39].

In the GDNS group, actual energy targets were underestimated, consistent with another report [40]. REE is frequently elevated in cancer patients due to tumour growth and inflammation [41]. While performed in most of the patients, ICal results were not consistently applied to guide the determination of energy targets. This may reflect dietitians’ reliance on predictive equations in daily practice. The additional 30% activity factor is a common approach in the Netherlands but may overlook interindividual differences [42].

The GDNS group demonstrated better QoL compared to usual care, in line with previous findings [33]. Although one-year survival rates were consistent with previous findings, GDNS did not affect these rates [43]. Unexpectedly, greater intraoperative blood loss, longer surgery duration and hospital stay, and higher sepsis incidence occurred in GDNS than in usual care. This may reflect a learning curve effect in the GDNS group, where robot-assisted surgery was introduced during the study period, whereas in usual care this was already implemented [44].

### Strengths and limitations

The prospective design allowed for systematic data collection and close monitoring through repeated nutritional assessment measurements, providing a comprehensive overview of changes throughout the treatment trajectory. Furthermore, the inclusion of a control hospital strengthened the study’s generalisability and clinical relevance. Limitations include the reduced sample size, due to patient dropout and the challenges associated with study performance during the COVID-19 pandemic. Detailed nutritional data were not collected in usual care due to logistical reasons, as care was provided across multiple locations, limiting assessment of protocol adherence.

Although a comprehensive nutritional assessment was performed by dietitians at predefined study visits, detailed information on the frequency, duration, and content of dietitian consultations was not systematically collected. As dietitians were not blinded to group allocation, the risk of performance bias in the intervention hospital cannot be excluded. Physical activity was not assessed during the study period, therefore their potential confounding effect on functional and muscle mass outcomes could not be evaluated. Due to later inclusion, usual care may have evolved to resemble the GDNS approach more closely. The research nurse may have fulfilled the role of a case manager, enabling earlier recognition and response to changes in patient condition, similar to the GDNS group.

Although group-specific differences in perioperative care remained, including a higher use of early oral feeding and shorter nasogastric tube duration in the usual care group, both the GDNS and usual care groups adhered to ERAS principles by providing early postoperative enteral nutrition. Residual confounding cannot be completely excluded; nevertheless, we consider it unlikely that differences in oral feeding alone explain the observed long-term differences in ASMI change after surgery. Furthermore, although CT is considered the gold standard for assessing skeletal muscle mass, access was limited in the usual care group, precluding direct comparisons of CT-derived muscle mass between groups. Therefore, BIA-ASMI was used and showed significant correlations with CT-SMI and urinary creatine in the GDNS group. This supports its use as a surrogate measure of skeletal muscle mass, consistent with previous findings [45].

### Clinical implications

The observed decline-recovery-decline pattern in muscle mass aligns with a prior study, supporting our findings [26]. Notably, GDNS appeared to attenuate declines in nutritional status more effectively than usual care, highlighting the importance of nutritional management. Our findings indicate that predicted energy requirements may underestimate measured energy expenditure. However, further studies are required to evaluate the clinical benefit of ICal-guided nutritional support. Furthermore, as both nutrition and physical activity play crucial roles in muscle maintenance, integrated interventions are important to optimise patient recovery [34]. However, substantial heterogeneity in treatment courses calls for personalised care, particularly in the postoperative phase [34, 46]. This requires easy access to dietetic and physical therapy services and close collaboration between primary and tertiary care [47].

## Conclusion

This study demonstrates that both GDNS and usual care, ASMI declines during CRT, improves after CRT, and then decreases again following oesophagectomy. The magnitude of ASMI loss is smaller in the GDNS group than in usual care during CRT and at 12 months postoperatively, although these differences do not reach statistical significance. GDNS is associated with more favourable outcomes in nutritional intake, body composition, and quality of life, but a higher sepsis rate and no survival benefit compared to usual care. Our findings underscore the need for personalised, integrated interventions to optimise recovery, particularly postoperatively.

## Supplementary Information

Below is the link to the electronic supplementary material.

Supplementary file 1 (DOCX 81 KB)

Supplementary file 2 (DOCX 722 KB)

Supplementary file 3 (DOCX 33 KB)

## Data Availability

The data that support the findings of this study are available up on reasonable request from the corresponding author. The data are not publicly available due to privacy restrictions.

## Declarations

### Visualisation

Figures and pictograms were created using PowerPoint, BioRender.com, and icons from Flaticon.com.
