# Supplementary material for: Goal-directed nutritional support in preserving muscle mass and optimising recovery in treatment of oesophageal cancer: results of a prospective non-randomised cluster trial
Source: Support Care Cancer. 2026 Sep 3;34(10):929. doi: 10.1007/s00520-026-11153-4 (PMC13541950; doi:10.1007/s00520-026-11153-4)
Supplement: Supplementary file 1 [file 520_2026_11153_MOESM1_ESM.docx]

**Supplement 1: Methods**

**Goal-directed nutritional support in preserving muscle mass and optimising recovery in treatment of oesophageal cancer: results of a prospective non-randomised cluster trial**

Iris Barth, Iris Stelwagen, Linda BM Weerink, Dorienke Gort-van Dijk, Hanna Meinders, Milos Milovanovic, Jan Willem Haveman, Marc J van Det, Gerard Dijkstra, Marjo JE Campmans-Kuijpers.

**Corresponding author:**

Iris Barth (e-mail: i.barth@umcg.nl).

**Content**

1. Intervention protocol: general information, research visits and dietetic consultation
2. Energy and protein requirements, nutritional intake
3. Indirect calorimetry
4. Nutritional assessment

4.1 Bio-electrical impedance vector analyser

4.2 Anthropometry

4.3 Functional outcomes

4.4 Malnutrition screening and sarcopenia

5. Urinary creatinine

##### 1. **Intervention protocol: general information, research visits and dietetic consultation**

- The main goal is that the patient receives optimal nutritional support throughout the entire treatment trajectory, including during chemoradiotherapy, hospital admission, and for one year after admission. As a general principle, nutritional care is provided by the same dietitian throughout the treatment trajectory, and nutritional intake is monitored.
- Dietetic consultations need to be scheduled prior to CRT (baseline, V0), during chemoradiotherapy (V1-V2), after completion of chemoradiotherapy and before surgery (V3), before hospital discharge (S1), and at 3, 6 and 12 months after surgery (S3-S12). Additional consultations can be scheduled when clinically indicated, based on nutritional intake, weight loss, nutrition-related symptoms, or the need for adjustment of nutritional support.
- Nutritional counselling aims to achieve individual energy and protein requirements and prevent deterioration of nutritional status throughout the treatment trajectory. Energy and protein intake should not fall more than 10% below the recommended amount. Patients should receive instructions on how to assess body weight at home. The nutritional assessment measurements, calculation of energy- and protein requirements should be performed according to the UMCG protocols (detailed in section 2-5).

*Preoperatively*

- Accordingly, the patient is offered oral nutritional supplements as soon as possible. The patient receives a selection of oral nutritional supplements with different flavours to try.
- If the patient’s nutritional requirements are still not met, enteral tube feeding is initiated. In this case, the surgeon is contacted as soon as possible to arrange an appointment at the endoscopy centre.
- If adequate nutritional intake cannot be achieved with enteral tube feeding, parenteral nutrition is initiated as soon as possible.
- At each visit, the PG-SGA is completed to provide immediate insight into the symptoms and nutrition-related problems experienced by the patient. Even in the absence of symptoms, the dietitian contacts the patient at least three times during chemoradiotherapy to monitor nutritional status and document energy and protein intake.
- For up to three months after surgery, the patient is asked to record their daily dietary intake. During follow-up contacts, the dietitian assesses the patient’s dietary intake.
- Energy requirements are determined with indirect calorimetry. If indirect calorimetry cannot be performed, predictive equations are used. An additional 30% is added to account for physical activity.

*Postoperatively*

- During oesophageal resection, a feeding jejunostomy is placed. Enteral tube feeding via the jejunostomy is initiated immediately after surgery. If the patient is clinically stable and/or following a contrast swallow study, the nasogastric tube may be removed. Following removal of the nasogastric tube, clear liquids are initiated. The following day, a full liquid diet is introduced, and on postoperative day 3, the patient can resume a regular diet including (supplemented) enteral tube feeding. If enteral nutrition is contraindicated, parenteral nutrition is administered via a peripherally inserted central catheter (PICC). In case of a chyle leak, a Medium Chain Triglyceride (MCT) diet and elemental tube feeding (e.g. Peptisorb® or Vivonex®) are initiated. If the chyle leak persists, total parenteral nutrition (TPN) is initiated.
- Early post-operatively, follow-up will take place every two weeks whenever possible (including the research visits), either in person or by telephone, to allow timely adjustment of nutritional support. The patient and/or dietitian record body weight every two weeks during the first three months.

##### 2. **Energy and protein requirements, nutritional intake**

In both the goal-directed nutritional support (GDNS) group and usual care, total energy expenditure (TEE) was predicted using resting energy expenditure (REE) via either the World Health Organization (WHO) or Harris-Benedict formula [1], with an additional 30% for disease and physical activity. Protein requirements were set at 1.2-1.5 grams per kilogram bodyweight [g/kg], using adjusted weight for BMI<20 or>30 (BMI=20 and 27.5 respectively). The use of oral nutritional supplements and EN/tube feeding was reported, including the type and quantity. For the GDNS group, dietary energy intake [kcal] and protein intake [g] were calculated and expressed as a percentage of the estimated targets and in intake per kg bodyweight.

##### 3. **Indirect calorimetry**

In the GDNS group, ICal was conducted to measure REE based on oxygen consumption (VO_2_ in l/min) and carbon dioxide production (VCO_2_ in l/min) via a ventilated hood (Quark RMR, COSMED). The respiratory quotient (RQ) was calculated as the ratio of VCO_2_ to VO_2_, with an expected standard value of ≈ 0.8 [2,3]. A percentage of 30% for disease and physical activity was added to the REE to calculate TEE.

4. **Nutritional assessment**

4.1 Bio-electrical impedance vector analyser

An 8-point bio-electrical impedance vector analyser (BIA) measured the body’s resistance (R) [Ω] and reactance (Xc) [Ω] to a weak alternating current of 50-60 kilohertz with multiple frequencies (Seca mBCA 525) [4]. Next, based on the impedance (Z), age, gender, height, body weight, waist circumference, the body composition was estimated: fat free mass index (FFMI), fat mass index (FMI), and phase angle (PhA). Appendicular skeletal muscle mass (ASM) was calculated according to the following formula by *Kyle* et. al [5]:

$ASM [kg]= -4.211+\left( 0.267\times RI \right)+\left( 0.095*weight [kg] \right)+\left( 1.909*sex \right)+\left( -0.01\times*age \right)+(0.058\times Xc)$

In this formula, RI is the resistance index (height [cm]^2^/resistance[Ω]), and sex is either man (1) or woman (0). To obtain appendicular skeletal muscle index (ASMI), ASM was divided by height squared: ASMI [kg/m^2^] = ASM [kg/height[m^2^].

4.2 Anthropometry

Height [cm] was measured in a standing position with a wall-mounted stadiometer. Body weight [kg] was measured to the nearest 0.1 kg using a digital weighing scale, without shoes and wearing light clothing. Body mass index (BMI) was calculated as body weight [kg] divided by squared height [m^2^]. Other anthropometric measurements included: waist-circumference (WC), mid-upper arm circumference (MUAC), triceps skinfold (TSF), and mid-upper arm muscle circumference (MAMC). Using a flexible, non-stretch measuring tape, WC was measured at the midpoint between the top of the iliac crest and the lower margin of the last palpable ribs. MUAC was determined in millimetres using the same measuring tape placed at the midpoint between the acromion on the shoulder blade and the olecranon on the ulna. Next, the posterior surface of this midpoint was used to pull the skin slightly with the thumb and forefinger, to place a calliper on the skinfold (Harpender® skinfold calliper) [6]. The mean of three TSF [mm] measurements was reported. Next, MAMC [mm^2^] was calculated by: MUAC - [TSF*3.14]) [7].

4.3 Functional measures

The maximum handgrip strength (HGS) was measured with a hand dynamometer (JamarTM®) in kilograms (kg), with the highest of three attempts used in the analyses.

4.4 Malnutrition screening and sarcopenia

To screen for malnutrition risk, the PG-SGA was filled out by the patient (part 1, short form; PG-SGA SF) and the dietitian (part 2). The PG-SGA SF was utilised in usual care, and the complete PG-SGA (part 1 and 2) in GDNS. Score ranges are 0-36 ( PG-SGA SF) and 0-52 ( complete PG-SGA), including categorisation of patients as well-, moderately/suspected, or severely malnourished (stage A, B, and C respectively). Additionally, malnutrition was defined using the Global Leadership Initiative on Malnutrition (GLIM) criteria, which requires at least one phenotypic (non-volitional weight loss, low BMI, reduced muscle mass) and one etiologic criterion (reduced food intake, disease burden) [8]. In our study, non-volitional weight loss was assessed with the PG-SGA SF, while low muscle mass and low BMI were defined using previously described ASMI cut-offs [9] and age-specific BMI thresholds (<20 kg/m² for <70 years; <22 kg/m² for ≥70 years). Sarcopenia was determined based ASMI cut-offs combined with low HGS (<27 kg for men;<16 kg for women) [9–11].

5. **Urinary creatinine**

Before the next outpatient visit, the patient collected a 24-hour urine sample . The first morning urine on the day of collection was discarded. Thereafter, all urine was collected for 24 hours, including the first morning urine on the following day. Urine creatinine and total urine volume were subsequently measured.

**References**

1. Harris JA, Benedict FG. A Biometric Study of Human Basal Metabolism. *Proc Natl Acad Sci U S A*. 1918 Dec;4(12):370-3. doi: 10.1073/pnas.4.12.370.
2. Weir JB de V. New Methods for Calculating Metabolic Rate with Special Reference to Protein Metabolism. *J Physiol*. 1949 Aug;109(1-2):1-9. doi: 10.1113/jphysiol.1949.sp004363.
3. Nutritional Assessment Platform. SOP indirecte calorimetrie (EN). Accessed November 7, 2024. https://nutritionalassessment.nl/standaard-operating-procedure-english-versions/
4. Seca Medical Measuring Systems and Scales Hamburg https://www. seca. com/en_nl/products/body composition analysis/seca mbca advantages/seca mbca 525. html. Seca mBCA 525. Accessed June 10, 2023.
5. Kyle UG, Genton L, Hans D, Pichard C. Validation of a bioelectrical impedance analysis equation to predict appendicular skeletal muscle mass (ASMM). *Clin Nutr*. 2003;22(6):537-543. doi:10.1016/S0261-5614(03)00048-7.
6. Baty International WSU. Harpenden, skinfold caliper. http://www.harpenden-skinfold.com/index.html. Accessed June 10, 2023.
7. Frisancho AR. New norms of upper limb fat and muscle areas for assessment of nutritional status. *Am J Clin Nutr*. 1981;34(11):2540-2545. doi:10.1093/ajcn/34.11.2540.
8. Jensen GL, Cederholm T, Correia MITD, et al. GLIM Criteria for the Diagnosis of Malnutrition: A Consensus Report From the Global Clinical Nutrition Community. *JPEN J Parenter Enteral Nutr..* 2019;43(1):32-40. doi:10.1002/jpen.1440.
9. Cruz-Jentoft AJ, Bahat G, Bauer J, et al. Sarcopenia: revised European consensus on definition and diagnosis. *Age Ageing*. 2019;48(1):16-31. doi:10.1093/ageing/afy169.
10. Dodds RM, Syddall HE, Cooper R, et al. Grip strength across the life course: Normative data from twelve British studies. *PLoS One*. 2014;9(12). doi:10.1371/journal.pone.0113637.
11. Gould H, Brennan SL, Kotowicz MA, Nicholson GC, Pasco JA. Total and appendicular lean mass reference ranges for Australian men and women: The Geelong osteoporosis study. *Calcif Tissue Int*. 2014;94(4):363-372. doi:10.1007/s00223-013-9830-7.
