# Supplementary material for: Goal-directed nutritional support in preserving muscle mass and optimising recovery in treatment of oesophageal cancer: results of a prospective non-randomised cluster trial
Source: Support Care Cancer. 2026 Sep 3;34(10):929. doi: 10.1007/s00520-026-11153-4 (PMC13541950; doi:10.1007/s00520-026-11153-4)
Supplement: Supplementary file 2 [file 520_2026_11153_MOESM2_ESM.docx]

**Supplement 2: Tables and Figures.**

**Goal-directed nutritional support in preserving muscle mass and optimising recovery in treatment of oesophageal cancer: results of a prospective non-randomised cluster trial**

Iris Barth, Iris Stelwagen, Linda BM Weerink, Dorienke Gort-van Dijk, Hanna Meinders, Milos Milovanovic, Jan Willem Haveman, Marc J van Det Gerard Dijkstra, Marjo JE Campmans-Kuijpers.

**Corresponding author:**

Iris Barth (e-mail: i.barth@umcg.nl).

**Content**

**Supplementary tables:**

**Table S1:** Surgical and tumour characteristics

**Table S2**: Analysis of covariance for the difference in appendicular skeletal muscle index change

**Table S3**: Generalised linear mixed effects model analysis of appendicular skeletal muscle index

**Table S4**: CT-muscle measurements for the goal-directed nutritional support group

**Table S5**: Postoperative complications

**Table S6**: Energy and protein targets, intake, and indirect calorimetry data for the goal-directed nutritional support group

**Table S7**: Nutritional support characteristics

**Table S8**: Nutritional assessment measurements

**Table S9**: Screening and assessment of malnutrition and sarcopenia

**Supplementary figures:**

**Figure S1**: Predicted appendicular skeletal muscle index trajectories over time

**Figure S2**: One-year overall- and disease-free survival

**Figure S3**: Quality of life according to the EORTC-QLQ-C30

# **Table S1** | Surgical and tumour characteristics

| **Surgical/post-operative characteristics** | **GDNS (n=36)** | **Usual care (n=39)** |
| --- | --- | --- |
| ASA classification |  |  |
| I | 1 (2.8) | 1 (2.6) |
| II | 27 (75.0) | 18 (46.2) |
| III | 7 (19.4) | 0 |
| *Missing* | *1 (2.8)* | *20 (51.3)* |
| Type of surgery^a^ |  |  |
| Open | 1 (2.8) | 0 |
| (RA)MIE | 35 (97.2) | 37 (94.9) |
| Other | 0 | 1 (2.6) |
| Position anastomosis |  |  |
| Cervical | 2 (5.6) | 2 (5.1) |
| Thoracal | 34 (94.4) | 37 (94.9) |
| Surgical procedure duration [minutes] | 474±84.1^**^ | 390±61.6 |
| Blood loss [ml] | 110 [70, 200] ^*^ | 100 [73, 100] |
| Nasogastric tube | 36 (100.0) | 35 (89.7) |
| Duration nasogastric tube [days] | 7.0 [6.0, 11.5] ^**^ | 4.0 [3.0, 5.0] |
| Postoperative feeding tube |  |  |
| Jejunostomy | 36 (100.0) | 38 (97.4) |
| No feeding tube | 0 | 1 (2.6) |
| Length of hospital admission [days] | 13 [9, 17] ^*^ | 7 [7, 8] |
| **Tumour characteristics** | **GDNS (n=36)** | **Usual care (n=39)** |
| Resected lymph nodes | 30 [21, 37] | 25 [22, 31] |
| ypTN classification |  |  |
| T(umour) |  |  |
| 0 | 2 (5.6) | 8 (20.5) |
| 1 | 7 (19.4) | 5 (12.8) |
| 2 | 6 (16.7) | 7 (17.9) |
| 3 | 20 (55.6) | 17 (43.6) |
| 4 | 0 | 2 (5.1) |
| N(odes) |  |  |
| 0 | 19 (52.8) | 18 (46.2) |
| 1 | 9 (25.0) | 13 (33.3) |
| 2 | 7 (19.4) | 6 (15.4) |
| Mandard tumour regression grade (TRG) ^b^ |  |  |
| TRG1 | 3 (8.3) | 3 (7.7) |
| TRG2 | 3 (8.3) | 11 (28.2) |
| TRG3 | 16 (44.4) | 11 (28.2) |
| TRG4 | 10 (27.8) | 9 (23.1) |
| *Missing* | *4 (11.1)* | *5 (12.8)* |
| Continuous variables are presented as mean±standard deviation or median [IQR], and categorical variables as frequencies (N) with percentages (%). Missing data were reported when ≥10%. Abbreviations/symbols: ASA, American Society of Anaesthesiology classification (I=normal/healthy. II, mild systemic disease. III, severe systemic disease. IV, severe systemic disease, life threatening). (RA)MIE, (robot-assisted) minimally invasive oesophagectomy. ^a^ Type of surgery: open, laparotomy/thoracotomy. (RA)MIE, laparoscopy/thoracoscopy. Other, cardia-oesophageal resection. ^b^ TRG1, complete regression (absence of residual cancer and fibrosis). TRG2, presence of rare residual cancer cells scattered through the fibrosis. TRG3, increase in number of residual cancer cells (fibrosis still predominated). TRG4, residual cancer outgrowing fibrosis. ^*^*P*<0.05. ^**^*P*<0.001. | | |

# **Table S2** | Analysis of (co)variance for the difference in appendicular skeletal muscle index change

|  | **ANOVA** | | | | **ANCOVA** | | | | |  |
| --- | --- | --- | --- | --- | --- | --- | --- | --- | --- | --- |
| **(A) CRT completion** | **ꞵ** | **SE** | **95%CI** | ***P*-value** | **ꞵ** | **SE** | **95%CI** | ***P*-value** | | |
| Group (hospital):  Usual care (intercept)  GDNS | -3.20  0.68 | 0.83  1.27 | -4.85 to -1.56  -1.85 to 3.21 | <0.001^**^  0.595 | 2.92  0.08 | 5.69  1.35 | -8.42 to 14.3  -2.61 to 2.76 | 0.609  0.955 | | |
| **Univariable analyses** |  | | | |  |  |  |  | | |
| Smoking status:  Former  Current | -0.21  0.81 | 1.49  2.86 | -3.17 to 2.74  -4.89 to 6.50 | 0.886  0.778 | 0.07  0.55 | 1.48  2.83 | -2.88 to 3.02  -5.10 to 6.20 | 0.962  0.847 | | |
| Phase angle [º] | -0.48 | 0.79 | -2.05 to 1.08 | 0.542 | -1.44 | 0.96 | -3.35 to 0.47 | 0.138 | | |
| Handgrip strength [kg] | 0.06 | 0.05 | -0.04 to 0.16 | 0.242 | 0.10 | 0.06 | -0.02 to 0.23 | 0.108 | | |
| Body mass index [kg/m^2^] | -0.09 | 0.14 | -0.37 to 0.20 | 0.555 | -0.08 | 0.15 | -0.37 to 0.21 | 0.598 | | |
| Sex [female] | -2.25 | 1.66 | -5.56 to 1.05 | 0.179 |  |  |  |  | | |
| Age [years] | -0.00 | 0.07 | -0.14 to 0.13 | 0.941 |  |  |  |  | | |
| **(B) Preoperatively** | **ꞵ** | **SE** | **95%CI** | ***P*-value** | **ꞵ** | **SE** | **95%CI** | ***P*-value** | | |
| Group (hospital):  Usual care (intercept)  GDNS | -0.58  -0.23 | 1.10  1.86 | -2.80 to 1.63  -3.95 to 3.50 | 0.601  0.904 | 3.70  -0.68 | 7.31  2.14 | -11.0 to 18.4  -4.99 to 3.63 | 0.615  0.752 | | |
| **Univariable analyses** |  | | | |  |  |  |  | | |
| Smoking status:  Former  Current | 4.31  5.55 | 2.00  3.93 | 0.28 to 8.33  -2.36 to 13.5 | 0.036  0.164 | 4.62  5.33 | 2.12  3.96 | 0.34 to 8.89  -2.66 to 13.3 | 0.035^*^  0.186 | | |
| Phase angle [º] | -1.18 | 1.08 | -3.35 to 0.99 | 0.279 | -0.88 | 1.38 | -3.65 to 1.89 | 0.525 | | |
| Sex [female] | -3.64 | 2.70 | -9.07 to 1.79 | 0.184 | -5.30 | 3.09 | -11.5 to 0.94 | 0.094 | | |
| Handgrip strength [kg] ^a^ | -0.03 | 0.07 | -0.18 to 0.12 | 0.697 | -0.04 | 0.11 | -0.25 to 0.18 | 0.738 | | |
| Age [years] | 0.01 | 0.09 | -0.18 to 0.19 | 0.948 |  |  |  |  | | |
| Body mass index [kg/m^2^] | -0.11 | 0.22 | -0.55 to 0.33 | 0.617 |  |  |  |  | | |
| **(C) After surgery** | **ꞵ** | **SE** | **95%CI** | ***P*-value** | **ꞵ** | **SE** | **95%CI** | ***P*-value** | | |
| Group (hospital):  Usual care (intercept)  GDNS | -7.09  1.34 | 1.60  3.03 | -10.4 to -3.78  -4.92 to 7.60 | <0.001^**^  0.662 | -14.4  3.34 | 13.2  3.89 | -42.3 to 13.6  -4.87 to 11.6 | 0.293  0.402 | | |
| **Univariable analyses** |  |  |  |  |  |  |  |  | | |
| LOS [days] | -0.06 | 0.24 | -0.55 to 0.43 | 0.812 | -0.23 | 0.39 | -1.06 to 0.59 | 0.556 | | |
| Smoking status:  Never  Former | Ref  -2.67 | 3.0 | -8.82 to 3.48 | 0.377 | Ref  -1.44 | 3.43 | -8.69 to 5.81 | 0.680 | | |
| Handgrip strength [kg] ^b^ | 0.06 | 0.15 | -0.24 to 0.36 | 0.696 | 0.13 | 0.21 | -0.32 to 0.58 | 0.554 | | |
| Age [years] | 0.10 | 0.13 | -0.17 to 0.36 | 0.455 | 0.06 | 0.14 | -0.24 to 0.37 | 0.665 | | |
| **(D) After surgery** | **ꞵ** | **SE** | **95%CI** | ***P*-value** | **ꞵ** | **SE** | **95%CI** | ***P*-value** | | |
| Group (hospital):  Usual care (intercept)  GDNS | -7.09  1.34 | 1.60  3.03 | -10.4 to -3.78  -4.92 to 7.60 | <0.001^**^  0.662 | -3.25  2.17 | 17.5  4.00 | -33.1 to 41.0  -6.27 to 10.6 | 0.825  0.595 | | |
| **Univariable analyses** |  | | | |  |  |  |  | | |
| LOS [days] | -0.06 | 0.24 | -0.55 to 0.43 | 0.812 | -0.25 | 0.38 | -1.06 to 0.57 | 0.531 | | |
| Sepsis | -6.53 | 6.83 | -20.6 to 7.60 | 0.349 | -7.16 | 7.91 | -23.9 to 9.51 | 0.377 | | |
| Smoking status:  Never  Former | Ref  -2.67 | 3.0 | -8.82 to 3.48 | 0.377 | Ref  -1.85 | 3.36 | -8.93 to 5.26 | 0.589 | | |
| Handgrip strength [kg] ^b^ | 0.06 | 0.15 | -0.24 to 0.36 | 0.696 | 0.14 | 0.21 | -0.31 to 0.58 | 0.520 | | |
| Sex [female] | -0.63 | 3.72 | -8.32 to 7.06 | 0.867 |  |  |  |  | | |
| Body mass index [kg/m^2^] | 0.20 | 0.42 | -0.66 to 1.06 | 0.638 |  |  |  |  | | |
| Surgical duration [minutes] | 0.00 | 0.02 | -0.04 to 0.04 | 0.894 |  |  |  |  | | |
| Age [years] | 0.10 | 0.13 | -0.17 to 0.36 | 0.455 |  |  |  |  | | |
| Abbreviations and symbols: ANOVA, analysis of variance. ANCOVA, analysis of covariance. GDNS, goal-directed nutritional support protocol. SE, standard error. β, beta-coefficient for the independent variable in the model. 95%CI, 95% confidence interval. LOS, length of hospital stay. AR^2^, adjusted R-squared. ^a^ At baseline (V0). ^b^ After chemo-radiotherapy /before surgery (V3). ^*^*P*<0.05. ^**^*P*<0.001.  (A) Analyses of the change in ASMI [%] between visit 0 (baseline) and visit 2 (3-6 weeks after start chemoradiotherapy). Model statistics: AR^2^ -0.029. *P*=0.711. F-statistic: 0.6232 on 6 and 74 DF.  (B) Analyses of the change in ASMI [%] between visit 0 (baseline) and visit 3 (2-4 months after start chemoradiotherapy/before surgery) as the outcome. Model statistics: AR^2^ -0.061. *P*=0.190. F-statistic: 1.534 on 6 and 43 DF.  (C) Analyses of the change in ASMI [%] between visit 3 (2-4 months after start chemoradiotherapy/before surgery) and the 12 month follow-up visit after surgery, as the outcome. Model statistics: AR^2^ -0.202. *P*=0.928. F-statistic: 0.2607 on 5 and 17 DF.  (D) Sensitivity analysis (analysis C, including sepsis as a covariable). Model statistics: AR2 -0.160, P=0.845. F-statistic: 0.3947 on 5 and 17 DF. | | | | | | | | |  |  |

# **Table S3** | Generalised linear mixed effects model analysis of appendicular skeletal muscle index

| **Unadjusted interaction^ab^** | **ꞵ** | **SE** | **95%CI** | **t value** |  |
| --- | --- | --- | --- | --- | --- |
| Intercept (usual care) | 7.27 | 0.15 | 6.98, 7.55 | 4.89 |  |
| Time (linear) | 0.02 | 0.01 | 0.00, 0.03 | 2.43 |  |
| Time (quadratic) | 0.00 | 0.00 | 0.00, 0.00 | 3.18 |  |
| GDNS | -0.14 | 0.22 | -0.58, 0.29 | -0.65 |  |
| Time (linear) x GDNS | 0.01 | 0.02 | -0.03, 0.05 | 0.35 |  |
| Time (quadratic) x GDNS | 0.00 | 0.00 | -0.00, 0.00 | 0.41 |  |
| **Adjusted interaction^ac^** | **ꞵ** | **SE** | **95%CI** | **t value** |  |
| Intercept (usual care) | 8.08 | 0.65 | 6.79, 9.37 | 12.4 |  |
| Time (linear) | 0.02 | 0.01 | 0.00, 0.03 | 2.46 |  |
| Time (quadratic) | 0.00 | 0.00 | 0.00, 0.00 | 3.14 |  |
| GDNS | -0.09 | 0.19 | -0.48, 0.29 | -0.49 |  |
| Sex |  |  |  |  |  |
| Male | Ref |  |  |  |  |
| Female | -1.20 | 0.26 | -1.70, -0.69 | -4.66 |  |
| Smoking status |  |  |  |  |  |
| Never | Ref. |  |  |  |  |
| Former | -0.07 | 0.20 | -0.46, 0.31 | 0.38 |  |
| Current | -0.17 | 0.36 | -0.88, 0.54 | -0.48 |  |
| Handgrip strength (baseline) | 0.02 | 0.01 | 0.00, 0.03 | 1.71 |  |
| Time (linear) x GDNS | 0.01 | 0.02 | -0.03, 0.05 | 0.66 |  |
| Time (quadratic) x GDNS | 0.00 | 0.00 | 0.00, 0.00 | 0.69 |  |
| Abbreviations: GDNS, goal-directed nutritional support group (intervention). AIC, Akaike Information Criterion. ICC, intraclass correlation coefficient. Conditional R^2^, conditional R-squared (the variance explained by both fixed and random effects). Marginal R^2^, marginal R-squared (the variance explained by predictors in the model). ^a^ Generalised Linear Mixed effects Model (GLMM), including time $\times$ group interaction centered at week 17 after CRT. ^b^ Adjusted ICC, 0.905. Conditional R^2^, 0.906. Marginal R^2^, 0.011. AIC, 421.8. ^c^ Adjusted ICC, 0.855. Conditional R^2^, 0.906. Marginal R^2^, 0.354. AIC, 368.6. | | | | | |

# **Table S4** | CT-muscle measurements for the goal-directed nutritional support group

|  | **Start of**  **CRT** | **After CRT/**  **before surgery** | **12 months**  **after surgery** | **Change from**  **V0 to V3** | | | **Change from**  **V3 to S12** | | |  |
| --- | --- | --- | --- | --- | --- | --- | --- | --- | --- | --- |
| **Skeletal muscle index (SMI)** | **V0 (n=40)** | **V3 (n=25)** | **S12 (n=15)** | **∆** | **95%CI** | ***P*-value** | **∆** | **95%CI** | ***P*-value** |  |
| Low SMI ^a^ | 23 (56.1) | 14 (56.0) | 13 (86.7) |  |  |  |  |  |  |  |
| SMI [cm/m^2^]  Females  Males | 44.0 ±10.9  37.8±7.62  46.5±11.2 | 46.5±9.4  38.7±5.93  50.1±8.47 | 44.1±8.4  35.5±3.41  47.2±7.42 | -0.07  1.07  -0.55 | -0.32 to 3.08  -3.66 to 5.80  -4.93 to 3.82 | 0.965  0.587  0.789 | -6.21  -3.05  -7.39 | -11.8 to -0.63  -7.05 to 0.94  -15.4 to 0.62 | 0.032^*^  0.082  0.065 |  |
| **Psoas muscle index (PMI)** | **V0 (n=43)** | **V3 (n=25)** | **S12 (n=15)** | **∆** | **95%CI** | ***P*-value** | **∆** | **95%CI** | ***P*-value** |  |
|  |  |  |  |  |  |  |  |  |  |  |
| Low PMI ^b^ | 8 (18.5) | 4 (23.5) | 6 (66.7) |  |  |  |  |  |  |  |
| PMI [cm/m^2^]  Females  Males | 6.68±1.91  5.20±0.98  7.25±1.88 | 6.58±1.96  5.33±1.09  7.17±2.03 | 6.01±1.93  4.98±1.25  6.39±2.04 | -0.11  0.35  -0.31 | -0.57 to 0.35  -0.32 to 1.02  -0.92 to 0.30 | 0.613  0.236  0.290 | -0.91  -0.20  -1.17 | -2.06 to 2.25  -0.38 to -0.02  -2.83 to 0.48 | 0.111  0.041^*^  0.138 |  |
| Continuous data are presented as mean±standard deviation and categorical data as frequencies (N) with percentages (%). Abbreviations and symbols: CRT, chemoradiotherapy. V0, baseline visit prior to CRT. V3, visit 3 (2-4 months after start CRT/CT before surgery). S12, 12 months after surgery. n=, number of observations. ^a.^ Low SMI is defined as: <41 cm^2^/m^2^ for females and < 43 cm^2^/m^2^ for males with BMI < 25 kg/m^2^ and SMI < 53 cm^2^/m^2^ for males with BMI ≥ 25 kg/m^2^ (28). ^b.^ Low PMI is defined as: <4.11 cm^2^/m^2^ for females and <5.65 cm^2^/m^2^ for males (29). ^*^*P*<0.05. | | | | | | | | | | |

# **Table S5** | Postoperative complications

|  | **Before**  **discharge** | | **6 months postoperative** | | **12 months postoperative** | |
| --- | --- | --- | --- | --- | --- | --- |
|  | **GDNS**  **(n=36)** | **Usual care**  **(n=39)** | **GDNS**  **(n=28)** | **Usual care**  **(n=25)** | **GDNS**  **(n=16)** | **Usual care**  **(n=21)** |
| Complication rate: | 25 (69.4) | 22 (56.4) |  |  |  |  |
| Clavien Dindo grade:^a^ |  |  |  |  |  |  |
| I | - | - |  |  |  |  |
| II | 11 (44.0) | 15 (68.2) |  |  |  |  |
| III | 9 (36.0) | 3 (13.6) |  |  |  |  |
| IV | 8 (32.0) | 2 (9.1) |  |  |  |  |
| V | 1 (0.04) | 2 (9.1) |  |  |  |  |
| Recurrent nerve paresis | 2 (5.7) | 1 (2.6) | 1 (3.6) | 0 | 0 | 1 (4.8) |
| Pleural empyema | 1 (2.8) | 3 (7.7) | 1 (3.6) | 0 | 0 | 0 |
| Pneumonia | 10 (27.8) | 7 (17.9) | 0 | 0 | 0 | 0 |
| Mediastinal abscess | 1 (2.8) | 0 (0.0) | 0 | 0 | 0 | 0 |
| Cardiac complications | 10 (27.8) | 8 (20.5) | 2 (7.1) | 1 (4.0) | 1 (6.3) | 0 |
| Chyle leak | 3 (8.3) | 4 (10.3) | 1 (3.6) | 0 | 0 | 0 |
| Delayed gastric emptying | 3 (8.3) | 2 (5.1) | 4 (14.3) | 0 | 3 (5.3) | 0 |
| Anastomotic leakage | 9 (25.0) | 3 (7.7) | 4 (14.3) | 0 | 0 | 0 |
| Wound infection | 0 | 0 | 1 (3.6) | 0 | 1 (6.3) | 0 |
| Wound dehiscence | 0 | 0 | 0 | 0 | 0 | 0 |
| Reintervention ^b^ | 9 (25.0) | 4 (10.3) | 8 (28.6) | 1 (4.0) | 10 (62.5) | 4 (19.0) |
| Reoperation | 4 (11.1) | 3 (7.7) | - | 0 | 1 (6.3) | 1 (4.8) |
| Sepsis | 7 (19.4) ^*^ | 1 (2.6) | 1 (3.6) | 0 | 2 (12.5) | 0 |
| ICU readmission | 4 (11.1) | 3 (7.7) | 1 (3.6) | 0 | 1 (6.3) | 1 (4.8) |
| Reintubation | 5 (13.9) | 3 (7.7) | 1 (3.6) | 0 | 1 (6.3) | 0 |
| Organ failure | 4 (11.1) | 1 (2.6) | 1 (3.6) | 0 | 3 (5.3) | 0 |
| Death | 0 | 0 | 2 (7.1) | 6 (24.0) | 5 (31.3) | 1 (4.8) |
| Hospital readmission |  |  |  |  | 15 (93.8) | 8 (38.1) |
| Data are presented as frequencies (N) with percentages (%). Abbreviations/symbols: GDNS, goal directed nutritional support (intervention). ^a^ Complication severity by Clavien Dindo grading : I, any deviation from the normal postoperative course without the need for pharmacological treatment or surgical , endoscopic, and radiological interventions. II, requiring pharmacological treatment with drugs other than such allowed for grad I complications (including blood transfusions and total parenteral nutrition). III, requiring surgical, endoscopic or radiological intervention. IV, life-threatening complications (including central nervous system) requiring intermediate care or intensive care unit management. V, death of a patient. ^b^ Reinterventions included: GDNS: pyloric botox injection, liver biopsy, stent replacement, stenosis dilatation, duodenal tube placement, pleural puncture. Usual care: pyloric botox injection, gastroscopy for stenosis inspection, duodenal tube placement, stenosis dilatation. ^*^*P*<0.05 (compared to usual care). | | | | | | |

# **Table S6** | Energy and protein targets, intake, and indirect calorimetry data for the goal-directed nutritional support group

|  | **During chemoradiotherapy** | | | **After oesophagectomy** | | | |  |
| --- | --- | --- | --- | --- | --- | --- | --- | --- |
| **Energy** | **V0 (n=50)** | **V1 (n=49)** | **V2 (n=48)** | **S1 (n=36)** | **S3 (n=33)** | **S6 (n=27)** | **S12 (n=16)** |  |
| TEE [kcal] | 2392 [2037, 2600] | 2300 [2059, 2545] | 2289 [2074, 2500] | 2135 [2016, 2311] | 2152 [2017, 2331] | 2104 [1858, 2379] | 2139 [1990, 2229] |  |
| Intake [k] | 1875 ± 471 | 2142 ± 521 | 2232 ± 550 ^c^ | 2033 ± 540 ^c^ | 2176 ± 523 | 1853 ± 624 | 2126 ± 246 |  |
| Intake/BW [kcal/kg] | 23.4 ± 8.06 | NA | 28.0 ± 7.9 ^c^ | 24.4 ± 7.60 ^c^ | NA | NA | 28.8 ± 8.32 |  |
| target met [%] | 83.6 [69.9, 96.7] | 96.5 [80.5, 110] | 104 [76.8, 114] ^c^ | 94.1 [81.9, 108] | 101 [89.8, 118] | 91.1 [23.9, 102] | 95.0 [86.0, 112] |  |
| **Method energy target** | **V0 (n=34)** | **V1 (n=36)** | **V2 (n=42)** | **S1 (n=30)** | **S3 (n=28)** | **S6 (n=21)** | **S12 (n=16)** |  |
| H & B | 5 (10.0) | 7 (14.0) | 10 (20.0) | 1 (2.0) | 0 (0.0) | 3 (27.3) | 0 (0.0) |  |
| Indirect calorimetry | 11 (22.0) | 14 (28.0) | 18 (36.0) | 1 (2.0) | 0 (0.0) | 1 (2.0) | 0 (0.0) |  |
| WHO | 18 (36.0) | 15 (30.0) | 14 (28.0) | 28 (56.0) | 28 (96.6) | 17 (2.0) | 17 (34.0) |  |
| Other | 0 (0.0) | 0 (0.0) | 0 (0.0) | 0 (0.0) | 0 (0.0) | 0 (0.0) | 0 (0.0) |  |
| **Protein** | **V0 (n=50)** | **V1 (n=49)** | **V2 (n=48)** | **S1 (n=36)** | **S3 (n=33)** | **S6 (n=27)** | **S12 (n=16)** |  |
| Intake [g/day] | 82.7 ± 21.7 | 97.4 ± 26.5 | 105 ± 30.0 ^c^ | 95.6 ± 28.4 ^c^ | 99.8 ± 27.5 ^c^ | 75.8 ± 28.6 ^c^ | 81.8 ± 21.7 |  |
| Intake/BW [g/kg] | 1.03 ± 0.37 | NA | 1.32 ± 0.43 ^c^ | 1.15 ± 0.39 ^c^ | NA | NA | 1.09 ± 0.30 |  |
| Target (1.2g/kg) met [%] | 85.0 [72.2, 102] | 104 [85.6, 123] | 113 [93.4, 130] ^c^ | 108 [82.5, 122] ^c^ | 102 [92.7, 133] | 84.4 [60.6, 91.4] | 85.7 [71.4, 109] |  |
| Target (1.5g/kg) met [%] | 67.5 [57.0, 81.8] | 87.0 [68.1, 101] | 91.1 [80.1, 105] ^c^ | 87.0 [69.6, 97.4] ^c^ | 81.6 [74.0, 106] | 68.4 [56.5, 73.9] | 68.6 [54.3, 85.0] |  |
|  | **V0** | | **V2** | |  | | |  |
| **Indirect calorimetry** | **Measured ^a^** | **Predicted ^b^** | **Measured ^a^** | **Predicted ^b^** |  |  |  |  |
| ICal performed | 44 (88.0) | NA | 18 (37.5) | NA |  |  |  |  |
| REE [kcal] | 1854 [1703, 2060] ^d^ | 1680 [1425, 1820] ^d^ | 1862 [1634, 2012] ^d^ | 1602 [1452, 1750] ^d^ |  |  |  |  |
| REE [kcal/kg] | 22.6 [21.3, 25.1] ^d^ | 19.2 [18.3, 21.3] ^d^ | 21.9 [18.9, 25.4] ^d^ | 19.8 [17.8, 21.8] ^d^ |  |  |  |  |
| TEE (REE+30%) [kcal] | 2410 [2214, 2677] | 2392 [2037, 2600] | 2421 [2124, 2616] | 2289 [2074, 2500] |  |  |  |  |
| RQ [VCO_2_/ VO_2_] | 0.91 [0.83, 0.95] |  | 0.92 [0.83, 1.02] |  |  |  |  |  |
| VO_2_ [ml/min] | 264 [237, 294] |  | 264 [230, 282] |  |  |  |  |  |
| VCO_2_ [ml/min] | 238 [223, 265] |  | 245 [209, 284] |  |  |  |  |  |
| *Missing* | *6 (12.0)* |  | *14 (28.0)* |  |  |  |  |  |
| Continuous outcomes are presented as mean±standard deviation, mean[interquartile range], and categorical outcomes as frequencies (N) with percentages (%). Abbreviations/symbols: V0, baseline. V1, visit 1 (1 week after start CRT). V2, visit 2 (3-6 weeks after start CRT). S1, early postoperatively/before discharge. S3, 3 months after oesophagectomy. S6, 6 months after oesophagectomy. S12, 12 months after oesophagectomy. ICal, indirect calorimetry. NA, not applicable. BW, bodyweight. Kcal/day, absolute energy intake [kcal] per day. Kcal/kg, energy expressed in kcal per kilogram body weight. Kcal/FFM, energy expressed in kcal per kilogram fat free mass. REE, resting energy expenditure. TEE, total energy expenditure predicted with equations and a 30% addition for activities (REE + 30%). RQ, respiratory quotient. VO_2_, consumed oxygen. VCO_2_, produced carbon dioxide. ^a^ Measured with indirect calorimetry. ^b^ Predicted with either Harris & Benedict formula or WHO formula. ^c^ *P*<0.05 within-group change: V2 (change between V1 and V2), S1 (change between V2 and S1), S3 (change between S1 and S3), S6 (change between S3 and S6). ^d^ *P*<0.05 between-group difference: measured compared to predicted (measured REE [kcal/day] compared to predicted REE [kcal/day], at V0 and V2). | | | | | | | | |

# **Table S7** | Nutritional support characteristics

| **During chemoradiotherapy** | | | | | **Postoperative** | | | | | | |
| --- | --- | --- | --- | --- | --- | --- | --- | --- | --- | --- | --- |
|  |  | |  | | **Early oral feeding postoperative (day 1 & 3)** | | | |  | |  |
|  |  | |  | | **Usual care** | | **GDNS** | |  | |  |
|  |  |  |  |  | **Total (n)** | **Oral feeding (n)** | **Total (n)** | **Oral feeding (n)** |  |  |  |
|  |  |  |  |  | Day 1: 37 | 30 (81.1) | 34 | 0 (0) |  |  |  |
|  |  |  |  |  | Day 3: 37 | 35 (94.6) | 32 | 0 (0) |  |  |  |
| **Oral nutritional supplements/**  **sip feeds** | **V0** | | **V2** | | **S1** | | **S3** | | **S6** | | **S12** |
|  | **Usual care**  **(n=49)** | **GDNS**  **(n=40)** | **Usual care**  **(n=42)** | **GDNS**  **(n=46)** | **Usual care**  **(n=34)** | **GDNS**  **(n=33)** | **Usual care**  **(n=32)** | **GDNS**  **(n=31)** | **Usual care**  **(n=15)** | **GDNS**  **(n=22)** | **GDNS**  **(n=16)** |
| ONS use | 18 (36.7) | 21 (52.5) | 26 (61.9) | 24 (52.2) | 32 (94.1) ^*^ | 3 (9.1) ^*^ | 19 (59.4) | 11 (35.5) | 5 (33.3) | 9 (40.9) | 6 (37.5) |
| ONS type: |  |  |  |  |  |  |  |  |  |  |  |
| *En+* | 3 (16.7) | 2 (9.5) | 3 (11.5) | 0 | 0 | 0 | 2 (10.5) | 0 | 1 (20.0) | 2 (22.2) | 0 |
| *En+ Pr-* | 1 (5.6) | 0 | 2 (7.7) | 0 | 6 (18.8) | 0 | 4 (21.1) | 0 | 1 (20.0) | 0 | 0 |
| *En+ Pr+* | 7 (38.9) | 8 (38.1) | 4 (15.4) | 3 (12.5) | 1 (3.1) | 0 | 1 (5.3) | 3 (27.3) | 0 | 1 (11.1) | 0 |
| *Conc* | 1 (5.6) | 0 | 3 (11.5) | 1 (4.2) | 2 (6.3) | 0 | 0 | 0 | 0 | 0 | 0 |
| *Conc F* | 0 | 4 (19.0) | 0 | 12 (50.0) | 0 | 0 | 0 | 1 (9.1) | 1 (20.0) | 1 (11.1) | 2 (33.3) |
| *Conc Pr+* | 5 (27.8) | 4 (19.0) | 10 (38.5) | 2 (8.3) | 20 (62.5) | 1 (33.3) | 9 (4.7) | 3 (27.3) | 2 (40.0) | 2 (22.2) | 0 |
| *Conc En+* | 0 | 1 (4.8) | 0 | 1 (4.2) | 0 | 0 | 0 | 0 | 0 | 0 | 0 |
| *Disease specific* | 0 | 1 (4.8) | 0 | 3 (12.5) | 0 | 0 | 0 | 1 (9.1) | 0 | 0 | 1 (16.7) |
| *Modules* | 0 | 0 | 1 (3.8) | 0 | 1 (3.1) | 2 (66.7) | 0 | 3 (27.3) | 0 | 3 (33.3) | 3 (50.0) |
| *Other* | 1 (5.6) | 1 (4.8) | 3 (11.5) | 1 (4.2) | 2 (6.3) | 0 | 3 (15.8) | 0 | 0 | 0 | 0 |
| ONS units: |  |  |  |  |  |  |  |  |  |  |  |
| *1* | 5 (27.8) | 4 (19.0) | 3 (11.5) | 10 (41.7) | 1 (3.1) | 1 (33.3) | 10 (52.6) | 6 (54.5) | 3 (60.0) | 4 (44.4) | 1 (16.7) |
| *2* | 8 (44.4) | 2 (9.5) | 12 (46.2) | 6 (25.0) | 17 (53.1) | 1 (33.3) | 7 (36.8) | 2 (18.2) | 2 (40.0) | - | 1 (16.7) |
| *3* | 2 (11.1) | 9 (42.9) | 3 (11.5) | 4 (16.7) | 7 (21.9) | 0 | 2 (10.5) | 1 (9.1) | 0 | 2 (22.2) | 2 (33.3) |
| *4* | 0 | 3 (14.3) | 3 (11.5) | 2 (8.3) | 0 | 0 | 0 | 0 | 0 | 1 (11.1) | 0 |
| *5* | 1 (5.6) | 1 (4.8) | 0 | 1 (4.2) | 0 | 0 | 0 | 0 | 0 | 1 (11.1) | 0 |
| *6* | 6 (33.3) | 2 (9.5) | 1 (3.8) | 0 | 0 | 0 | 0 | 0 | 0 | 0 | 0 |
| **Enteral and parenteral nutrition** | **V0** | | **V2** | | **S1** | | **S3** | | **S6** | | **S12** |
|  | **Usual care**  **(n=50)** | **GDNS**  **(n=50)** | **Usual care**  **(n=48)** | **GDNS**  **(n=48)** | **Usual care**  **(n=39)** | **GDNS**  **(n=35)** | **Usual care**  **(n=34)** | **GDNS**  **(n=33)** | **Usual care**  **(n=27)** | **GDNS**  **(n=28)** | **GDNS**  **(n=16)** |
| EN use | 5 (10.0) | 8 (8.0) | 11 (22.0) | 13 (26.0) | 28 (56.0) | 32 (64.0) | 18 (36.0) | 14 (28.0) | 1 (2.0) | 1 (2.0) | 1 (2.0) |
| EN type: |  |  |  |  |  |  |  |  |  |  |  |
| *En+ F* | 1 (20.0) | 0 | 2 (18.2) | 1 (7.7) | 0 | 1 (3.1) | 0 | 0 | 0 | 0 | 0 |
| *En+ Pr+* | 1 (20.0) | 0 | 1 (9.1) | 0 | 22 (78.6) | 0 | 13 (72.2) | 0 | 1 (100.0) | 0 | 0 |
| *En+ Pr+ F* | 2 (40.0) | 3 (37.5) | 5 (55.6) | 10 (76.9) | 0 | 27 (84.4) | 1 (5.6) | 14 (100.0) | 0 | 1 (100.0) | 0 |
| *St F* | 0 | 1 (12.5) | 0 | 1 (7.7) | 0 | 0 | 0 | 0 | 0 | 0 | 0 |
| *St Comp F* | 1 (20.0) | 0 | 0 | 0 | 0 | 0 | 0 | 0 | 0 | 0 | 0 |
| *Disease-specific* | 0 | 0 | 0 | 0 | 6 (21.4) | 3 (9.4) | 2 (11.1) | 0 | 0 | 0 | 0 |
| *Other* | 0 | 0 | 1 (9.1) | 0 | 0 | 0 | 0 | 0 | 0 | 0 | 0 |
| EN [ml] | 1500  [1375,1500] | 1650  [1375,1850] | 1500 ^*^  [1250,1590] | 2000 ^*^  [1800,2000] | 1000 ^*^  [1000,1000] | 1500 ^*^  [1000,2000] | 1000  [500,1000] | 1000  [1000,1000] | 1000  [1000, 1000] | 500  [500,500] | 1500  [1500,1500] |
| PN use |  |  |  |  | 5 (12.8) | 2 (5.7) |  |  |  |  |  |
| PN [days] |  |  |  |  | 15.0  [9.5, 20.5] | 4.00  [3.50, 4.50] |  |  |  |  |  |
| Continuous outcomes are presented as mean±standard deviation or median [interquartile range], and categorical variables as frequencies (N) with percentages (%). Abbreviations: GDNS, goal-directed nutritional support (intervention). V0, baseline. V1, visit 1 (1 week after start CRT) . V2, visit 2 (3-6 weeks after start CRT). S1, early postoperatively/before discharge. S3, 3 months after oesophagectomy. S6, 6 months after oesophagectomy. S12, 12 months after oesophagectomy. ONS, Oral Nutritional Supplements. EN, Enteral nutrition/tube feeding. PN, parenteral nutrition. WHO, World Health Organization. Conc, low volume/concentrated. Conc F, low volume/concentrated with fibre. Conc Pr+, low volume/concentrated protein enriched. Conc En+, low volume/concentrated energy enriched. En+, energy enriched. En+ F, energy enriched with fibre. En+ Pr-, energy enriched low in protein. En+ Pr+, energy and protein enriched. En+ Pr+ F, energy and protein enriched with fibre. St F, standard with fibre.  St Comp F, standard low volume with fibre. ^*^ *P*<0.05 (GDNS vs usual care) | | | | | | | | | | | |

# **Table S8** | Nutritional assessment measurements (anthropometry and bio-electrical impedance analysis)

|  | **During chemoradiotherapy** | | | | | **After oesophagectomy** | | | | |
| --- | --- | --- | --- | --- | --- | --- | --- | --- | --- | --- |
|  | **Visit** | **n** | **Usual care** | **n** | **GDNS** | **Visit** | **n** | **Usual care** | **n** | **GDNS** |
| Body mass  index (BMI)  [kg/m^2^] | V0 | 50 | 26.2±4.23 | 50 | 26.7±4.19 | S1 | 30 | 24.5±3.17 | 20 | 26.1±3.50 |
|  | V2 | 49 | 25.2±4.37 | 39 | 26.4±4.20 | S12 | 20 | 23.6±3.35 | 12 | 23.7±3.30 |
|  | V3 | 33 | 25.9±3.61 | 21 | 27.5±4.56 | ∆S1-V3 | 27 | -1.11±1.31 ^ab^ | 10 | 0.40±0.82 ^a^ |
|  | ∆V2-V0 | 49 | -1.01±0.84 ^ab^ | 39 | -0.57±0.85 ^ab^ | ∆S12-S1 | 18 | -1.04±1.34 ^b^ | 9 | -2.13±1.56 ^b^ |
|  | ∆V3-V2 | 33 | 1.20±0.99 ^b^ | 19 | 0.86±1.33 ^b^ |  |  |  |  |  |
| Waist circumference  (WC) [cm] | V0 | 50 | 99.5±12.2 | 49 | 99.1±13.4 | S1 | 30 | 96.3±11.3 | 20 | 102±10.8 |
|  | V2 | 49 | 97.7±12.3 | 38 | 100±13.5 | S12 | 20 | 91.9±12.4 | 13 | 91.9±12.5 |
|  | V3 | 36 | 101±18.6 | 20 | 103±12.1 | ∆S1-V3 | 27 | -4.93±18.5 | 10 | 1.10±3.48 |
|  | ∆V2-V0 | 49 | -2.10±4.62 ^ab^ | 38 | 0.84±5.72 ^a^ | ∆S12-S1 | 17 | -3.35±8.54 | 6 | -9.17±8.21 ^b^ |
|  | ∆V3-V2 | 34 | 5.03±15.6 | 18 | 1.78±4.71 |  |  |  |  |  |
| Triceps skinfold  thickness (TSF) [mm] | V0 | 50 | 12.1±3.3 ^a^ | 49 | 16.4±5.7 ^a^ | S1 | 27 | 12.8±4.3 | 26 | 14.8±4.9 |
|  | V2 | 48 | 12.7±3.3 ^a^ | 41 | 15.3±5.9 ^a^ | S12 | 21 | 12.3±3.36 | 14 | 15.4±5.63 |
|  | ∆V2-V0 | 48 | 0.69±4.07 | 40 | -1.34±6.53 | ∆S12-S1 | 18 | -0.89±5.72 | 9 | -2.26±7.01 |
| Mid-upper arm  muscle circumference  (MAMC)[mm^2^] | V0 | 50 | 268±40.0 | 48 | 261±33.0 | S1 | 27 | 268±34.7 | 26 | 255±37.6 |
|  | V2 | 48 | 263±35.6 | 41 | 257±30.8 | S12 | 21 | 258±38.5 | 14 | 248±34.4 |
|  | ∆V2-V0 | 48 | -7.41±23.5 ^b^ | 39 | -8.53±33.7 | ∆S12-S1 | 18 | -1.64±36.4 | 9 | 1.42±26.8 |
| Fat free mass index  (FFMI) [kg/m^2^] | V0 | 50 | 20.0±2.35 | 40 | 19.7±2.77 | S1 | 32 | 19.2±2.43 | 19 | 19.8±2.47 |
|  | V2 | 48 | 19.5±2.57 | 37 | 19.2±2.30 | S12 | 20 | 18.8±2.32 | 13 | 18.4±2.58 |
|  | V3 | 34 | 20.0±2.40 | 21 | 20.2±4.04 | ∆S1-V3 | 29 | -0.67±1.19 ^b^ | 10 | -0.37±2.87 |
|  | ∆V2-V0 | 48 | -0.51±1.45 ^b^ | 35 | -0.71±0.81 ^b^ | ∆S12-S1 | 19 | -0.22±1.21 | 5 | -0.94±2.62 |
|  | ∆V3-V2 | 34 | 0.39±1.31 | 18 | 0.80±2.56 |  |  |  |  |  |
| Fat mass index  (FMI) [kg/m^2^] | V0 | 50 | 5.4 [4.3, 7.7] | 41 | 6.3 [5.0, 9.5] | S1 | 32 | 5.0 [3.9, 6.1] ^a^ | 19 | 6.6 [5.0, 8.5] ^a^ |
|  | V2 | 48 | 4.7 [3.9, 7.2] | 37 | 7.2 [5.1, 10.1] | S12 | 20 | 4.6 [3.4, 5.7] | 13 | 6.5 [4.6, 7.1] |
|  | V3 | 34 | 5.6 [4.5, 7.6] ^a^ | 21 | 6.8 [5.5, 10.2] ^a^ | ∆S1-V3 | 29 | -0.70 [-1.50, 0.50] ^b^ | 10 | -0.50 [-0.75, 0.45] |
|  | ∆V2-V0 | 48 | -0.30 [-1.32, 0.42] ^a^ | 35 | 0.20 [-0.15, 0.95] ^a^ | ∆S12-S1 | 19 | -0.90 [-1.85, 0.05] | 5 | -0.20 [-0.60, 1.70] |
|  | ∆V3-V2 | 34 | 0.60 [-0.17, 1.85] ^b^ | 18 | 0.30 [-0.07, 1.58] |  |  |  |  |  |
| Phase angle  (PhA) [°] | V0 | 50 | 5.63±0.73 ^a^ | 41 | 6.00±0.82 ^a^ | S1 | 30 | 5.16±0.66 | 19 | 5.19±0.88 |
|  | V2 | 48 | 5.35±0.92 ^a^ | 37 | 5.84±0.80 ^a^ | S12 | 20 | 5.06±0.70 ^a^ | 12 | 5.60±0.70 ^a^ |
|  | V3 | 34 | 5.60±0.70 | 21 | 5.70±0.93 | ∆S1-V3 | 29 | -0.35±0.35 ^b^ | 10 | -0.45±0.49 ^b^ |
|  | ∆V2-V0 | 48 | -0.28±0.69 ^b^ | 35 | -0.22±0.52 ^b^ | ∆S12-S1 | 19 | -0.10±0.44 ^a^ | 5 | 0.64±0.69 ^a^ |
|  | ∆V3-V2 | 34 | 0.09±0.72 ^a^ | 18 | -0.41±0.50 ^ab^ |  |  |  |  |  |
| Handgrip strength  (HGS) [kg] | V0 | 49 | 39.7±10.9 ^a^ | 50 | 45.2±12.6 ^a^ | S1 | 21 | 40.6±9.4 | 26 | 40.9±10.6 |
|  | V2 | 45 | 40.8±10.4 ^a^ | 42 | 46.5±11.9 ^a^ | S12 | NA | NA | 14 | 43.7±8.6 |
|  | ∆V2-V0 | 44 | -0.39±6.36 | 42 | -0.45±4.96 | ∆S12-S1 | NA | NA | 9 | -1.00±3.91 |
| Continuous variables are presented as mean±standard deviation or median [interquartile range], and categorical variables as frequencies (N) with percentages (%). Abbreviations/symbols: GDNS, goal-directed nutritional support (intervention). V0, baseline. V1, visit 1 (1 week after start CRT). V2, visit 2 (3-6 weeks after start CRT). V3, visit 3 (2-4 months after start CRT/CT before surgery). S1, early postoperatively/before discharge. S3, 3 months after oesophagectomy. S6, 6 months after oesophagectomy. S12, 12 months after oesophagectomy. NA, not available. ^a^ Between-group difference (GDNS vs usual care); *P*<0.05. ^b^ Within-group change; *P*<0.05. | | | | | | | | | | |

# **Table S9** | Screening and assessment of malnutrition and sarcopenia

|  | **GDNS** | | | | | | **Usual care** | | | | | |
| --- | --- | --- | --- | --- | --- | --- | --- | --- | --- | --- | --- | --- |
|  | **During**  **CRT** | | | **After**  **oesophagectomy** | | | **During**  **CRT** | | | **After**  **oesophagectomy** | | |
| **PG-SGA** | **Visit** | **n** |  | **Visit** | **n** |  | **Visit** | **n** |  | **Visit** | **n** |  |
| Short form  (PG-SGA SF) | V0 | 49 | 5.1±4.9 | S1 | 26 | 7.4±3.5 | V0 | 32 | 4.2±3.5 | S1 | NA | NA |
|  | V1 | 47 | 4.9±5.1 | S3 | 27 | 5.4±3.4 | V1 | NA | NA | S3 | 25 | 5.6±4.3 |
|  | V2 | 40 | 7.9±5.9 ^a^ | S6 | 19 | 3.6±2.6 | V2 | 34 | 8.1±5.4 ^a^ | S6 | 26 | 5.1±3.6 |
|  |  |  |  | S12 | 16 | 3.1±2.7 |  |  |  | S12 | 20 | 3.0±2.6 |
| Full form | V0 | 48 | 7.1±5.3 | S1 | 24 | 9.2±3.7 |  | | | | | |
|  | V1 | 3 | 6.0±4.4 | S12 | 14 | 4.6±2.8 ^b^ |  |  |  |  |  |  |
|  | V2 | 33 | 10.7±6.3 |  |  |  |  |  |  |  |  |  |
| Malnutrition stage: |  |  |  |  |  |  |  |  |  |  |  |  |
| A. Well-nourished | V0 | 48 | 34 (68.0) | S1 | 25 | 0 |  |  |  |  |  |  |
|  | V1 | 4 | 2 (4.0) | S12 | 15 | 9 (18.0) |  |  |  |  |  |  |
|  | V2 | 36 | 15 (30.0) |  |  |  |  |  |  |  |  |  |
| B. Moderately malnourished | V0 | 48 | 11 (22.0) | S1 | 25 | 16 (32.0) |  |  |  |  |  |  |
|  | V1 | 4 | 1 (2.0) | S12 | 15 | 0 |  |  |  |  |  |  |
|  | V2 | 36 | 15 (30.0) |  |  |  |  |  |  |  |  |  |
| C. Severely malnourished | V0 | 48 | 4 (8.0) | S1 | 25 | 0 |  |  |  |  |  |  |
|  | V1 | 4 | 1 (2.0) | S12 | 15 | 0 |  |  |  |  |  |  |
|  | V2 | 36 | 4 (8.0) |  |  |  |  |  |  |  |  |  |
|  | **Visit** | **n** |  | **Visit** | **n** |  | **Visit** | **n** |  | **Visit** | **n** |  |
| **Malnutri-**  **tion^c^** | V0 | 41 | 14 (34.1) | S1 | 36 | 14 (38.9) | V0 | 48 | 17 (35.4) | S1 | 26 | 15 (57.7) |
|  | V2 | 41 | 24 (58.5) ^a^ | S12 | 28 | 8 (28.6) | V2 | 41 | 35 (85.4) ^a^ | S12 | 21 | 8 (38.1) |
| **Sarcopenia^d^** | V0 | 41 | - | S1 | 16 | 0 | V0 | 49 | 1 (2.0) | S1 | 33 | 0 |
|  | V2 | 37 | 1 (2.0) | S12 | 13 | 0 | V2 | 44 | 3 (6.8) | S12 | NA | NA |
| Continuous variables are presented as mean±standard deviation, and categorical variables as frequencies (N) with percentages (%).Abbreviations/symbols: CRT, chemoradiotherapy. NA, not available. GDNS, goal-directed nutritional support (intervention). PG-SGA (SF), Patient Generated-Subjective Global Assessment (Short Form); score range PG-SGA SF: 0-36, full PG-SGA range: 0-52.. V0, baseline. V1, visit 1 (1 week after start CRT). V2, visit 2 (3-6 weeks after start CRT). S1, early postoperatively/before discharge. S3, 3 months after oesophagectomy. S6, 6 months after oesophagectomy. S12, 12 months after oesophagectomy. ^a^ *P*<0.05 (within-group change between V0 and V2). ^b^ *P*<0.05 (within-group change between S1 and S12). ^c^ Criteria according to GLIM (Global Leadership Initiative on Malnutrition).^d^ Appendicular skeletal muscle index <7.0 kg/m² (men or <5.5 kg/m² (women), and a handgrip strength < 27 kg (men) or <16 kg (women). | | | | | | | | | | | | |

# **Supplementary Figures**

# **Figure S1** | Predicted appendicular skeletal muscle index trajectories over time. The black dotted line corresponds the centred timepoint week 17: the mean time between V0 (baseline) and V3 (after chemoradiotherapy) where ASMI was measured. The generalised linear mixed model includes the fixed effects for time (linear and quadratic), group (usual care and goal-directed nutritional support), and their interaction with random intercepts for subjects. Shaded areas represent 95% confidence intervals. Abbreviations: ASMI, appendicular skeletal muscle index

# **Figure S2** | One-year overall- and disease-free survival. Abbreviations: CRT, chemoradiotherapy. GDNS, goal-directed nutritional support group. OS, overall survival. DFS, disease-free survival. (A) OS after CRT and (B) esophagectomy. (C) DFS after CRT and (D) esophagectomy.

# **Figure S3** | Quality of life according to the EORTC-QLQ-C30. Abbreviations/symbols: EORTC-QLQ-C30, European Organization for Research and Treatment for Cancer Quality of Life Questionnaire (Score range: 0-100; higher score represents better health status/quality of life. GDNS, goal-directed nutritional support (intervention). QoL, quality of life. V0, baseline visit prior to CRT. V2, visit 2 (3-6 weeks after CRT initiation). S1, before discharge. S3/S12, 3 and 12 months after surgery. Scores: GDNS: V0, 83.3 [66.7, 100]. V2, 75.0^*^ [58.3, 83.3]. S1, 62.5 [43.4, 72.9]. S3, 75.0 [66.7, 83.3]. S12, 83.3 [66.7, 95.8]. Usual Care: 83.3 [72.9, 83.3]. V2, 66.7 [41.7, 75.0]. S1, 58.3 [41.7, 68.8]. S3, 75.0 [66.7, 83.3]. S12, 83.3 [75.0, 91.7]. **P*<0.05 (compared to usual care).
